# CD23 is a glycan-binding receptor in some mammalian species

**DOI:** 10.1074/jbc.RA119.010572

**Published:** 2019-09-05

**Authors:** Sabine A. F. Jégouzo, Hadar Feinberg, Andrew G. Morrison, Angela Holder, Alisha May, Zhiyao Huang, Linghua Jiang, Yi Lasanajak, David F. Smith, Dirk Werling, Kurt Drickamer, William I. Weis, Maureen E. Taylor

**Affiliations:** ‡ Department of Life Sciences, Imperial College London, London SW7 2AZ, United Kingdom; § Departments of Structural Biology and Molecular and Cellular Physiology, Stanford University School of Medicine, Stanford, California 94305; ¶ Department of Pathobiology and Population Sciences, Royal Veterinary College, North Mymms, Hatfield, Hertfordshire AL9 7TA, United Kingdom; ‖ Emory Comprehensive Glycomics Core, Emory University School of Medicine, Atlanta, Georgia 30322

**Keywords:** glycobiology, lectin, carbohydrate-binding protein, carbohydrate function, crystal structure, Fc receptor, carbohydrate-recognition domain, CLEC4J, FCER2 gene, glycan-binding receptors

## Abstract

CD23, the low-affinity IgE receptor found on B lymphocytes and other cells, contains a C-terminal lectin-like domain that resembles C-type carbohydrate-recognition domains (CRDs) found in many glycan-binding receptors. In most mammalian species, the CD23 residues required to form a sugar-binding site are present, although binding of CD23 to IgE does not involve sugars. Solid-phase binding competition assays, glycoprotein blotting experiments, and glycan array analysis employing the lectin-like domains of cow and mouse CD23 demonstrate that they bind to mannose, GlcNAc, glucose, and fucose and to glycoproteins that bear these sugars in nonreducing terminal positions. Crystal structures of the cow CRD in the presence of α-methyl mannoside and GlcNAcβ1–2Man reveal that a range of oligosaccharide ligands can be accommodated in an open binding site in which most interactions are with a single terminal sugar residue. Although mouse CD23 shows a pattern of monosaccharide and glycoprotein binding similar to cow CD23, the binding is weaker. In contrast, no sugar binding was observed in similar experiments with human CD23. The absence of sugar-binding activity correlates with accumulation of mutations in the gene for CD23 in the primate lineage leading to humans, resulting in loss of key sugar-binding residues. These results are consistent with a role for CD23 in many species as a receptor for potentially pathogenic microorganisms as well as IgE. However, the ability of CD23 to bind several different ligands varies between species, suggesting that it has distinct functions in different organisms.

## Introduction

CD23, also designated FcϵRII and CLEC4J, is a cell-surface receptor for the Fc domain of IgE, which regulates IgE synthesis (1). In addition to IgE, several other endogenous ligands have been proposed to bind to human CD23, including CD21, major histocompatibility locus class II receptors, and integrins α_M_β_2_ (CD11b–CD18), α_X_β_2_ (CD11c–CD18), α_V_β_3_ (vitronectin receptor), and α_V_β_5_ (2–4). These ligands interact with the extracellular, C-terminal portion of the receptor polypeptide. A portion of the extracellular domain can be released from the cell surface by proteolysis and can act on target cells by binding to ligands such as CD21 (2). The N-terminal cytoplasmic domain of the intact, membrane-bound form of CD23 also has multiple binding partners. In humans and mice, two isoforms of CD23, which result from differential splicing, have different N-terminal sequences (5). The presence of these different sequences correlates with the ability to mediate internalization of bound ligands, by endocytosis or phagocytosis, and to activate intracellular signaling pathways (4–6). One splice variant, designated CD23a, is expressed primarily on B lymphocytes, whereas variant CD23b is more broadly expressed on hematopoietic cells, including B and T lymphocytes, polymorphonuclear leukocytes, and follicular dendritic cells, as well as intestinal epithelial cells and bone marrow stromal cells.

The extracellular portion of CD23 consists of a C-terminal C-type lectin-like domain attached to the membrane by an extended neck region (1). The lectin-like domain is related to the C-type carbohydrate-recognition domains (CRDs)^3^ found in many mammalian glycan-binding receptors (7, 8). The neck region has been proposed to mediate formation of receptor trimers and is also implicated in binding to major histocompatibility locus class II receptors (3, 9). IgE and integrin binding have been mapped to different surfaces of the C-type lectin-like domain (10, 11), whereas binding of CD21 to a C-terminal extension is observed for human CD23, an interaction not conserved in mouse CD23 (12). Crystal structures of the C-terminal portion of human CD23 in complex with the Cϵ3 and Cϵ4 domains of IgE suggest a possible mode of interaction of the receptor with its immunoglobulin ligand, in which CD23 lectin-like domains bind to the Cϵ3 domains of the IgE dimer on the outside of a cavity formed by the Cϵ3 and Cϵ4 domains (10, 13). Binding to IgE is independent of the *N*-linked glycans attached to domain Cϵ3, which point into the cavity.

The C-type lectin-like domain of human CD23 resembles a sugar-binding domain, although there is conflicting evidence regarding the role of sugars in binding to different ligands. Recent studies with mouse CD23 show that it binds α-mannans and β-glucans from fungal cell walls, suggesting that CD23 from at least some species does have sugar-binding activity (14). Binding of sugars to C-type CRDs results from a common set of interactions between two sugar hydroxyl groups in a pyranose ring, a bound Ca^2+^, and a conserved set of amino acid side chains on the protein (7, 8). Many C-type lectin-like domains lack these conserved residues and interact with noncarbohydrate ligands, usually in a Ca^2+^-independent manner (7). Examples of such structurally and functionally distinct proteins include a family of natural killer cell receptors that bind major histocompatibility-related molecules and a receptor for oxidized low-density lipoprotein. Sequence comparisons indicate that these proteins lack the conserved Ca^2+^- and sugar-binding residues, and for the most part they form a distinct evolutionary group separate from the various groups of sugar-binding receptors. In contrast, the residues needed for sugar binding are conserved in mouse CD23, and sequencing of the *FCER2* gene from cow confirms that these residues are also present in cow CD23.^4^

Conservation of the sugar-binding residues prompted a re-evaluation of sugar binding of CD23. The results reported here demonstrate that CD23 from both cows and mice does bind to sugars in a Ca^2+^-dependent manner but that this activity has been lost in most primate species, including humans.

## Results

### Sugar-binding activity of cow CD23

**Figure 1.**
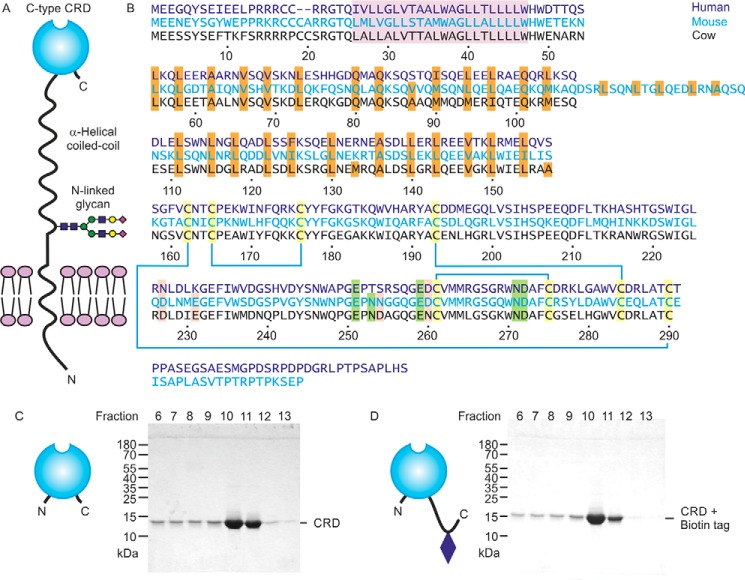
**Expression of cow CD23.** *A*, domain organization of CD23. *B*, sequence alignment of CD23 from human, mouse, and cow. The transmembrane region is *highlighted* in *violet*, and the *a* and *d* positions of the heptad repeats in the neck are *highlighted* in *orange*. Conserved cysteine residues, linked by disulfide bonds, are *shaded yellow*. Positions of residues that commonly interact with Ca^2+^ are indicated in *green* for the conserved sugar-binding site and in *pink* for an accessory site found in some C-type CRDs. Residue numbers correspond to the cow protein based on sequence XP_002688905.2 from the National Center for Biotechnology Information. *C* and *D*, SDS-PAGE of fractions eluted from a mannose-Sepharose column with EDTA for the CRD fragment alone and with an appended biotin tag. Gels were stained with Coomassie Blue.

The overall organization of CD23 proteins is summarized in Fig. 1*A*. An α-helical coiled-coil domain characterized by the presence of a heptad-repeat pattern of aliphatic hydrophobic amino acids in the stalk domain (Fig. 1*B*) mediates trimer formation. The globular C-terminal lectin-like domain is characterized by the presence of conserved cysteine residues that form disulfide bonds (Fig. 1*B*). The pattern of disulfide bonds is analogous to the arrangement of other C-type CRDs (10, 13, 15, 16). The alignment also reveals conservation of Ca^2+^ ligands in the mouse and cow proteins, but some of these residues are absent in the human protein.

Following protocols that have been used for purification of other C-type CRDs, a fragment of *Bos taurus* CD23 encompassing residues Ala^157^–Cys^290^, corresponding to the globular domain, was expressed in *Escherichia coli*. This segment of cow CD23 corresponds to the extensively characterized fragment of human CD23 that is produced by the Der P 1 protease from house mites, although cow CD23 lacks the C-terminal extension present in human CD23 (Fig. 1*B*) (10). Folded protein was obtained by dissolving inclusion bodies in guanidine and renaturation by dialysis in the presence of Ca^2+^. Sugar binding was tested by chromatography on a series of resins bearing immobilized monosaccharides, including mannose, GlcNAc, fucose, and galactose. Bound protein was eluted with EDTA. Binding was observed for the mannose and fucose resins (data for mannose shown; Fig. 1*C*). A significant amount of protein leaches off the column in the Ca^2+^-containing wash buffer that precedes the EDTA elution, indicating that binding is not as tight as for some CRDs, but the result indicates that cow CD23 does display Ca^2+^-dependent sugar-specific binding. To facilitate further analysis of the sugar-binding activity, an additional version of the CRD was expressed with a biotinylation sequence appended at the C-terminal end (Fig. 1*D*).

### Sugar-binding specificity of cow CD23

**Figure 2.**
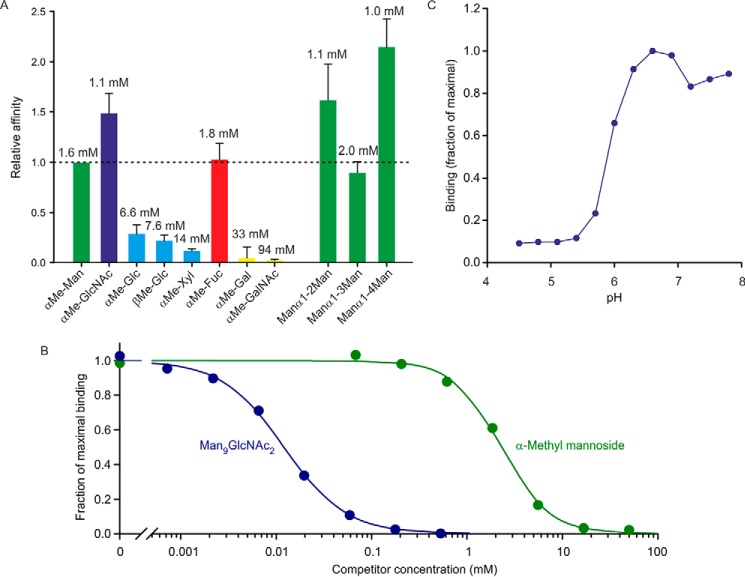
**Characterization of cow CD23 binding to sugars in a solid-phase binding assay.** *A*, binding competition assays in which immobilized CRD was probed with horseradish peroxidase, as reporter ligand, at concentrations well below saturation binding. Under these conditions, the relative *K_I_* values for competing monosaccharide and oligosaccharide ligands closely approximate *K_D_* values. *Solid bars*, *K_I_* values for various sugars relative to the *K_I_* for mannose. *Error bars*, S.D. for three or more assays for each sugar. Absolute *K_I_* values are indicated at the *top* of each *bar. B*, comparison of binding of α-methyl mannoside and Man_9_GlcNAc_2_ oligosaccharide. An example of a competition assay is shown. The average ratio of the affinities for five assays was 240 ± 50. *C*, pH dependence of binding was determined using the same solid-phase assay format.

The C-terminal biotinylation sequence was used to immobilize the CRD from cow CD23 in streptavidin-coated wells, which were probed using horseradish peroxidase as a reporter ligand. The mannose-terminated oligosaccharides on horseradish peroxidase make it a convenient ligand for mannose-binding CRDs that can be detected with a chromogenic substrate (17). Binding competition assays were used to compare the affinity of the CRD for a range of monosaccharides (Fig. 2*A*). The three monosaccharides with highest affinity are mannose, fucose, and GlcNAc, which is typical for many C-type CRDs that bind sugars with equatorial 3- and 4-OH groups (18). Several disaccharides were also tested, to see whether there was a strong preference for a particular linkage, but the affinities differed by at most 2-fold. However, the affinity for a Man_9_GlcNAc_2_ oligosaccharide was substantially enhanced (Fig. 2*B*).

The pH dependence of binding was also tested, demonstrating that the CRD releases ligand when the pH falls below 6 (Fig. 2*C*). This type of pH dependence is observed in recycling endocytic receptors that release their ligands at endosomal pH (19). The N-terminal sequence of the splice variant of CD23 shown in Fig. 1*A* contains the motif YSEF, which is analogous to the sequences YSEI in human CD23 and YSGY in mouse CD23. This motif represents a binding site for clathrin adapter protein 2, which is responsible for endocytosis of human and mouse CD23 expressed in B cells (6). Thus, the sequence of the cytoplasmic domain and the pH-dependent binding of sugar ligands are consistent with the suggestion that cow CD23 could mediate internalization of ligands based on a sugar-dependent recognition mechanism.

**Figure 3.**
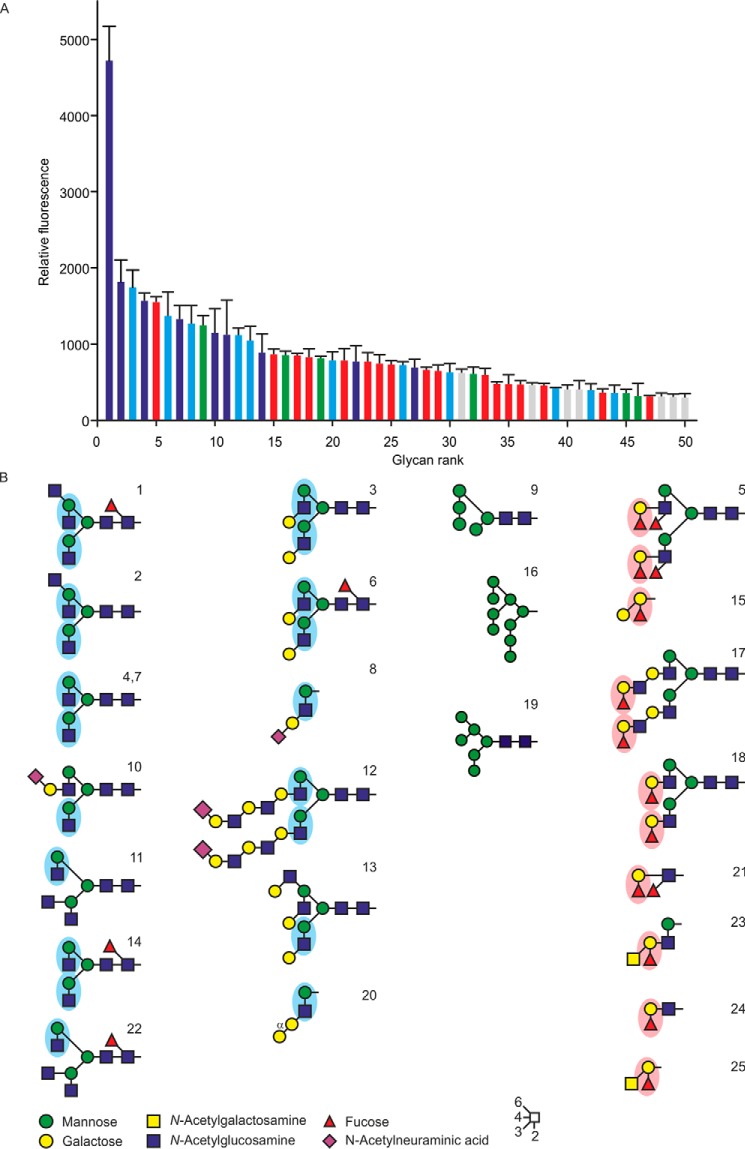
**Glycan array analysis of cow CD23.** *A*, a glycan array comprising oligosaccharides from the Consortium for Functional Glycomics was probed with a tetravalent complex of biotin-tagged CRD with fluorescently labeled streptavidin. Results are *color-coded* based on the presence of reducing-end GlcNAcβ1–2Man epitopes (*blue bars*), GlcNAcβ1–2Man with galactose linked 1–3 to the GlcNAc residue (*cyan bars*), oligomannose structures (*green bars*), or Fucα1–2Gal disaccharide (*red bars*). Complete results for all oligosaccharides on the array are given in Table S1. *B*, structures of oligosaccharide ligands that show the strongest signals on the glycan array. *Error bars*, S.D.

The ligand-binding specificity of cow CD23 was examined further by probing a glycan array with biotinylated CRD in complex with fluorescently labeled streptavidin (Fig. 3*A*). The results reveal binding to a wide range of glycan ligands, with a relatively broad and continuous distribution of intensities on the array, rather than highly selective binding to a single small class of ligands. However, some patterns can be observed (Fig. 3*B*). Most of the binding observed can be accounted for by the presence of one of three different epitopes. Many of the glycans that bind CD23 bear the disaccharide GlcNAcβ1–2Man, either exposed at a nonreducing terminus or with galactose residue appended to the 3-position of the GlcNAc residue. A second common epitope is a Fucα1–2Gal disaccharide, and several oligomannose structures are also bound.

Whereas these patterns suggest some degree of specificity beyond monosaccharide binding, it is important to note that other glycans that contain these structures do not bind as well, and the differences in affinity are relatively small. Thus, the binding may well reflect the similar affinities observed for mannose, GlcNAc, and fucose methyl glycosides, with different intensities of signals reflecting at least in part accessibility and abundance of these terminal residues.

**Figure 4.**
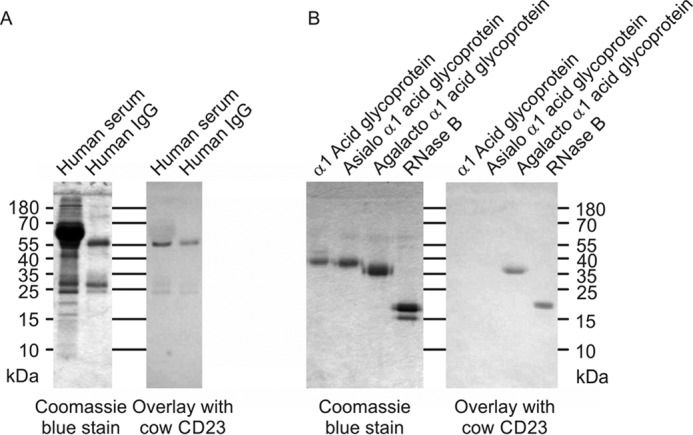
**Probing of glycoproteins with cow CD23.** Glycoproteins were separated by SDS-PAGE. In each *panel*, the Coomassie Blue–stained gel is shown on the *left*, and a blot probed with CRD-avidin-alkaline phosphatase complex is shown on the *right. A*, serum glycoproteins. *B*, natural glycoproteins and versions that have been modified to expose novel reducing-end sugars.

Similar observations were also made when glycoproteins were tested as ligands. Glycoproteins blotted onto nitrocellulose membranes were probed with CRD complexed with alkaline phosphatase–conjugated avidin complex (Fig. 4). The results in Fig. 4*A* demonstrate that the main serum ligand for CD23 appears to be IgG, which presumably reflects the presence of some exposed terminal GlcNAcβ1–2Man groups. The results are consistent with binding to oligomannose structures on RNase B (Fig. 4*B*). Exposure of terminal GlcNAcβ1–2Man groups on α_1_ acid glycoprotein dramatically enhances binding, although the natural protein is a poor ligand.

### Structural basis for sugar binding to cow CD23

**Figure 5.**
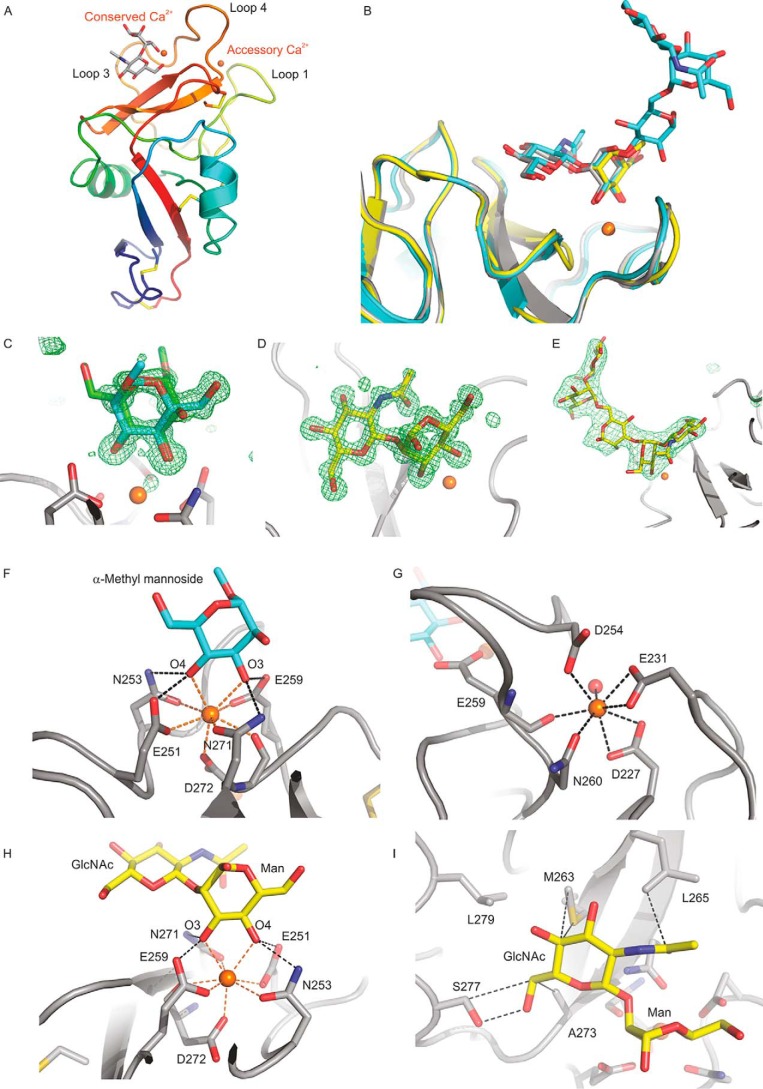
**Structure of the CRD from cow CD23.** *A*, overall structure of the CRD bound to GlcNAcβ1–2Man. The protein in a *cartoon representation* is *colored* from *blue* (N terminus) to *red* (C terminus). Disulfide bonds are shown in *yellow*. The disaccharide ligand is shown in a *stick representation*, with carbon atoms in *gray*, oxygen atoms in *red*, and Ca^2+^ in *orange. B*, superposition of ligand-binding sites in the three crystal structures, with carbon atoms shown in *yellow* for α-methyl mannoside, *gray* for GlcNAcβ1–2Man, and *cyan* for GlcNAc_2_Man_3_. Oxygen, nitrogen, and Ca^2+^ are as in *A. C–E*, *F_o_* − *F_c_* electron density maps calculated by omitting the sugar residue from the model, contoured at 3.0 σ, and shown as a *green mesh* for α-methyl mannoside (*C*), GlcNAcβ1–2Man (*D*), and GlcNAc_2_Man_3_ (*E*). *F–I*, *close-up views* of the Ca^2+^ and the ligand-binding sites. *F*, α-methyl mannoside in the primary sugar-binding site, with OH groups 3 and 4 of mannose ligated to the conserved Ca^2+^. For clarity, only one orientation of the sugar is shown. *G*, accessory Ca^2+^ site, with ligands from four side chains as well as the backbone carbonyl group of Glu^259^. *H*, GlcNAcβ1–2Man in the primary sugar-binding site, ligated to the conserved Ca^2+^. *I*, interactions of the GlcNAc moiety of GlcNAcβ1–2Man at the secondary binding site. The protein in *cartoon representation* as well as carbon atoms in *stick representations* are presented in *gray*, carbon atoms of the sugar are *cyan* for α-methyl mannoside and *yellow* for GlcNAcβ1–2Man, and other atoms are as in *A*.

The mechanism of sugar binding to cow CD23 was examined by X-ray crystallography. Crystals of the CRD from cow CD23 were obtained under conditions that allow binding of sugar ligands: in the presence of 5 mm Ca^2+^. Crystals obtained in the presence of α-methyl mannoside and GlcNAcβ1–2Man diffracted to 1.00 and 1.20 Å, respectively, and crystals with GlcNAc_2_Man_3_ oligosaccharide diffracted to 2.7 Å. The structure of the α-methyl mannoside complex was solved by molecular replacement, using the human CD23 structure (20) as the search model; this complex was then used to solve the other structures. The cow CD23 structure shows a typical C-type CRD fold (Fig. 5*A*), and the bound sugar ligands are well-defined (Fig. 5, *B–E*). The structure of cow CD23 confirms the presence of the four disulfide bonds predicted from the alignment with other C-type CRDs and also shows that there are two bound Ca^2+^, representing the conserved Ca^2+^ found at sugar-binding sites in C-type CRDs and the adjacent accessory site. The bound Ca^2+^ (Fig. 5, *F* and *G*) are ligated by the predicted residues that are highlighted in Fig. 1*A*. Several different crystal forms of the CRD from human CD23 have previously been characterized, two of which were obtained in the presence of Ca^2+^ (13, 20). These structures reveal the presence of only a single Ca^2+^, corresponding to the conserved principal site. The absence of the accessory Ca^2+^ correlates with changes at two of the positions that correspond to ligands for this Ca^2+^ in cow CD23.

Crystals obtained in the presence of the simple sugar ligands α-methyl mannoside and GlcNAcβ1–2Man showed that binding of mannose at the primary binding site results from coordination and hydrogen bonds with the 3- and 4-OH groups in the arrangement typically seen in C-type CRDs that bind mannose and related sugars (Fig. 5, *F* and *H*). The GlcNAc residue in the GlcNAcβ1–2Man disaccharide makes only a few additional contacts with the surface of the CRD (Fig. 5*I*). The surface near the primary binding site lacks obvious protrusions, so it is hard to define a secondary or extended binding site that would favor particular oligosaccharide ligands or occlude others. These observations are consistent with the relatively broad range of ligands bound in the glycan array experiment.

**Figure 6.**
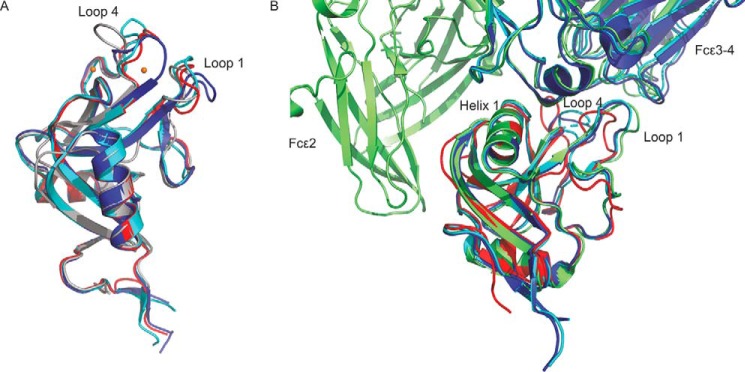
**Comparison of cow and human CD23.** *A*, flexibility in loops 1 and 4 shown in superposition of the CRDs of cow and human CD23. The CRD from the complex of cow CD23 with α-methyl mannoside is shown in *gray*. Human CD23 without bound Ca^2+^ (PDB entries 2H2R and 4G96) is shown in *blue* and *cyan*, and CD23 with Ca^2+^ bound is shown in *red* (PDB entry 2H2T). *B*, superposition of the CRD of cow CD23, with GlcNAcβ1–2Man in *red*, human CD23 complexed with domains Cϵ3 and Cϵ4 of IgE in the presence of Ca^2+^ (PDB entry 4GKO) in *blue*, human CD23 complexed with domains Cϵ3 and Cϵ4 of IgE in the absence of Ca^2+^ (PDB entry 4EZM) in *cyan*, and CD23 complexed with domains Cϵ2–Cϵ4 of IgE in the absence of Ca^2+^ (PDB entry 5LGK) in *green.*

When comparing cow CD23 and human CD23, the largest conformational differences are in two regions of the CRD that correspond to loops 1 and 4 (Fig. 6). The conformational differences are largely due to differences in Ca^2+^ binding. In previous work, it has been noted that these loops differ between human CD23 crystals obtained in the presence or absence of Ca^2+^ (13). Ca^2+^ binding in the primary site induces a 30-fold increase in affinity for IgE. The differences are seen both in the CRD structures and also in the complexes of human CD23 with IgE (Fig. 6). The Ca^2+^-bound conformations of human and cow CD23 differ from each other. In cow CD23, loops 1 and 4 are involved in binding both the conserved and the accessory Ca^2+^ site, whereas the latter site is missing in the human structure. Pro^252^ of cow CD23 is in the *cis* configuration typically found in C-type CRDs with bound Ca^2+^. Although the corresponding residue in human CD23, Pro^250^, is also in *cis* configuration, the conformation of the remainder of this loop is different from the cow structure, and there are significant differences in the Ca^2+^ ligands. In cow CD23, the side chains of Asn^253^ and Glu^259^ bind the conserved Ca^2+^, and the side chain of Asp^254^ binds the accessory Ca^2+^ that is absent in the human protein. The residue corresponding to Asp^254^ in cow CD23 is Ser^252^, which does not interact with Ca^2+^, and Glu^257^ in human CD23, corresponding to Glu^259^ in cow CD23, is a Ca^2+^ ligand in some of the crystal forms but not others.

The surface of human CD23 that interacts with domains Cϵ3 and Cϵ4 of IgE is largely conserved in the cow protein, specifically residues Trp^186^, Ile^187^, Gln^188^, Arg^190^, and Tyr^191^ in the first helix, and also residues Arg^226^ and Phe^274^. However, other interactions between human CD23 and IgE mediated by loops 1 and 4 (Fig. 6) could not occur with the cow protein, as these loops differ in sequence and conformation between human and cow CD23. Also, the surface of human CD23 that interacts with domain Cϵ2 of IgE does not seem to be conserved: the fold is similar but not the sequence.

**Figure 7.**
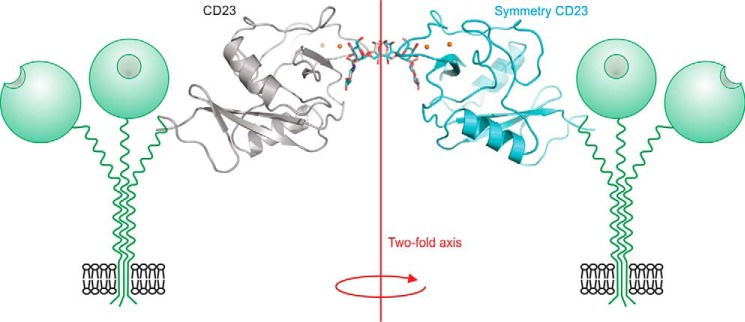
**Cross-linking of two CRDs of cow CD23 complexed with a biantennary ligand.** Overall orientation of CRDs bound to the two branches of the bi-antennary glycan is shown in the context of two receptor trimers. Individual terminal GlcNAcβ1–2Man disaccharides on two branches of the oligosaccharide interacting with sugar-binding sites in CRDs from CD23 and a symmetry-related molecule. The protein is shown in *cartoon representation* in *gray* and *cyan*. Ca^2+^ is represented in *orange spheres*. The biantennary ligand is shown in a *stick representation*, with carbon atoms in *gray* or *cyan* and oxygen atoms in *red*. The 2-fold axis relating the CRDs is perpendicular to the membrane.

An additional crystal form of the CRD from cow CD23 was obtained in complex with a biantennary glycan terminating in two GlcNAcβ1–2Man groups. The oligosaccharide bridges between CRDs related by a crystallographic 2-fold symmetry axis, so the electron density for the sugars represents an average of two possible orientations (Fig. 7). In each binding site, the terminal GlcNAcβ1–2Man residues occupy approximately the same positions as in the crystal with GlcNAcβ1–2Man disaccharide (Fig. 5*H*). Analysis using CARP and GlycoMapsDB shows that the observed torsion angles in the oligosaccharide are similar to other experimentally observed structures and to the favored conformations predicted from energy calculations (21, 22). Thus, it does not seem that the crystal packing distorts the oligosaccharide structure. The relative orientation of the two CRDs bound to a single oligosaccharide suggests a way in which binding to oligosaccharides could cross-link CRDs in two adjacent CD23 molecules on the surface of a cell, which represents a potential mechanism for initiating signaling (Fig. 7).

### Expression of CD23 on bovine B cells

**Figure 8.**
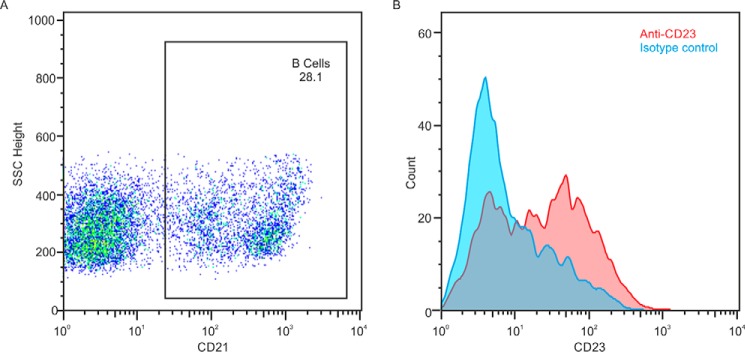
**Flow cytometry to demonstrate expression of cow CD23 on B cells.** Peripheral blood mononuclear cells were isolated from cow blood and stained with monoclonal anti-CD21 and anti-CD23 antibodies. *A*, gating of mature B cells based on forward and side scattering and CD21 expression. *B*, comparison of binding of antibody to CD23 and isotype control to the B cell population.

A previous screen of antibodies to human proteins against bovine tissues identified a commercially available mAb raised against human CD23 that cross-reacts with cow CD23 (23). The ability of the antibody to bind cow CD23 was verified by Western blotting. Flow cytometry of peripheral blood mononuclear cells isolated from bovine blood revealed expression of CD21^+^ B cells (Fig. 8), consistent with the pattern of human and mouse CD23 expression on mature circulating B cells.

### Mouse CD23 displays similar but distinct sugar-binding activity

**Figure 9.**
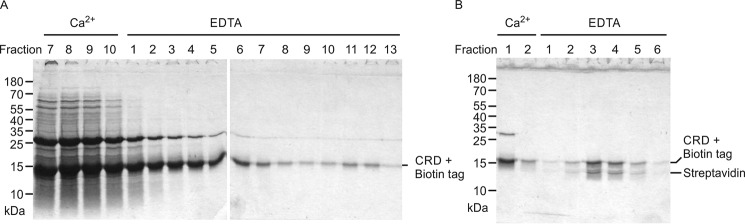
**Expression of mouse CD23.** *A*, SDS-PAGE of fractions eluted from a 10-ml mannose-Sepharose column for the CRD fragment of mouse CD23 with appended biotin tag. Following application of renatured protein, the column was washed with 10 1-ml aliquots of buffer containing 25 mm Ca^2+^ and eluted with 15 1-ml aliquots of buffer containing 2.5 mm EDTA. Fractions 11–12, corresponding to material weakly bound to the column, were used for coating of assay plates and formation of streptavidin complexes. *B*, SDS-PAGE of fractions eluted from a 1-ml mannose-Sepharose column for the biotin-tagged complexed with Alexa Fluor 647–labeled streptavidin. The column was washed with 1 ml of buffer containing 25 mm Ca^2+^ and eluted with seven aliquots (0.25 ml) of buffer containing 2.5 mm EDTA. Gels were stained with Coomassie Blue.

The amino acid residues that form the sugar-binding site in cow CD23 are conserved in mouse CD23 (Fig. 1*B*). Demonstration that cow CD23 has sugar-binding activity, as well as recent studies on binding of mouse CD23 to fungal cell wall polysaccharides (14), suggested that mouse CD23 would also bind sugars. A CRD fragment of mouse CD23, expressed with a C-terminal biotin tag in the same way as the cow protein, bound only weakly to the mannose-Sepharose resin, although retardation relative to other proteins was evident (Fig. 9*A*). Increased affinity was achieved by forming a tetrameric complex with streptavidin, which bound more tightly to the resin in the presence of Ca^2+^ and was then eluted with EDTA, confirming that mouse CD23 binds sugars in a Ca^2+^-dependent manner, although with lower affinity than cow CD23 (Fig. 9*B*).

**Figure 10.**
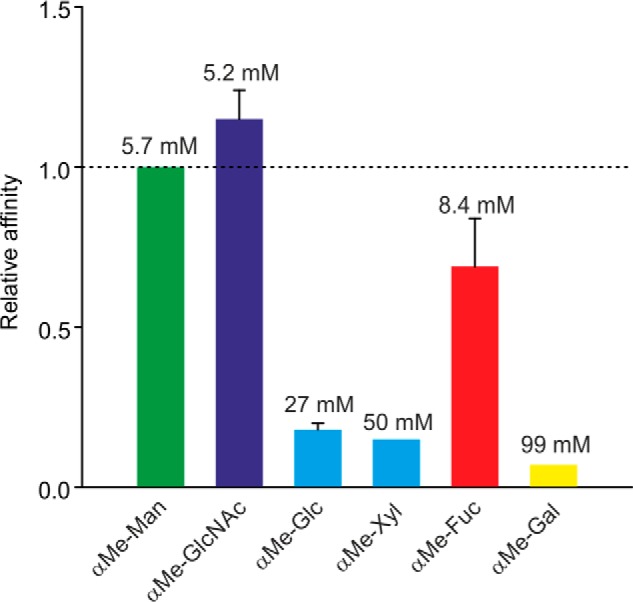
**Characterization of mouse CD23 binding to monosaccharides in solid-phase binding assay.** Binding competition assays were performed with immobilized CRD probed with horseradish peroxidase. *Error bars*, S.D. for three or more assays for each sugar. Absolute *K_I_* values are indicated at the *top* of each *bar*.

**Figure 11.**
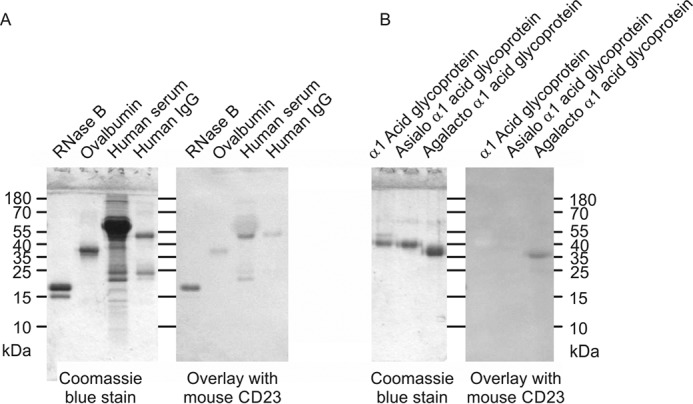
**Probing of glycoproteins with mouse CD23.** Glycoproteins were separated by SDS-PAGE. In each *panel*, Coomassie Blue-stained gel is shown on the *left*, and the blot probed with the CRD-avidin-alkaline phosphatase complex is shown on the *right. A*, natural glycoproteins. *B*, glycoproteins modified to expose novel reducing-end sugars.

Sugar binding was further characterized by applying biotin-tagged mouse CD23 to streptavidin-coated assay plates and probing these with horseradish peroxidase (Fig. 10). Binding competition assays with monosaccharides revealed binding to a similar range of sugars as for cow CD23, although the *K_I_* values for mouse CD23 are consistently higher, reflecting weaker binding to all of the sugars. Blotting experiments with the mouse CRD in complex with alkaline phosphatase–conjugated streptavidin also showed binding to a similar selection of glycoproteins as for cow CD23, including RNase B, with high-mannose oligosaccharides, and asialo, agalacto-α_1_-acid glycoprotein (Fig. 11). Attempts were also made to probe the glycan array with fluorescently labeled streptavidin-CRD complex. Because of the relatively weaker binding affinity, higher concentrations of complex were required to obtain signals. However, there were high background signals covering significant areas of the array surface, possibly reflecting protein aggregation, which resulted in positive signals for a wide range of oligosaccharides, and the results of several independent experiments did not correlate well. Thus, the arrays did not provide any further insights into the binding specificity of the mouse CD23.

### Absence of equivalent sugar-binding activity in human CD23

Similar fragments corresponding to the CRD from human CD23 failed to show any binding activity by affinity chromatography, even when biotin-tagged CRDs were complexed with streptavidin to increase the affinity (data not shown). These results correlate with the absence of one of the key Ca^2+^-ligating residues at the primary sugar-binding site (Fig. 1*A*). The importance of residue Asn^253^ is shown in the crystal structure of cow CD23, which confirms its role as both a coordination ligand for Ca^2+^ and a hydrogen bond donor that interacts with one of the sugar hydroxyl groups. The presence of a threonine residue at this position in human CD23 is thus consistent with the absence of detectable sugar-binding activity (13, 20).

**Figure 12.**
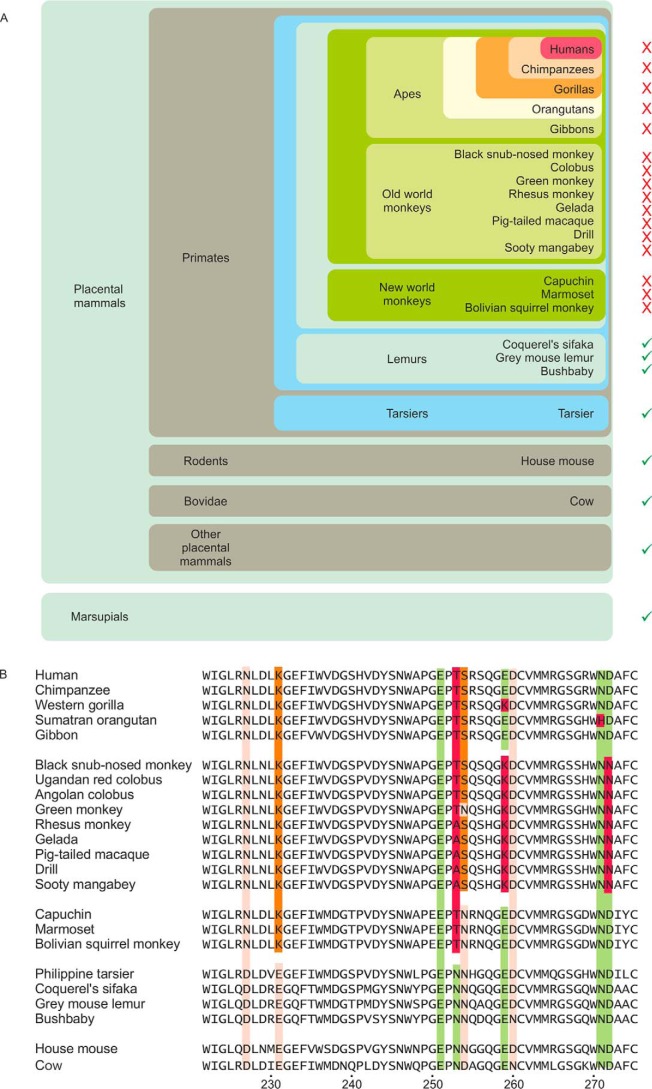
**Evolution of Ca^2+^- and sugar-binding sites in CD23.** *A*, monophyletic arrangement of groups of primates in which one or more of the residues needed to form functional Ca^2+^- and sugar-binding sites are mutated. For each genus, a *green check mark* indicates conservation of the sites compared with cow CD23, whereas a *red* × indicates that there are changes in one or more residues in these sites. *B*, sequence alignment showing details of amino acid changes in representative species within each genus. Sequences for all of the available mammalian CD23 orthologues are given in Fig. S1. Residues that ligate the conserved Ca^2+^ and sugar are indicated in *green*, and residues forming an accessory Ca^2+^ binding site are indicated in *pink*.

Phylogenetic analysis of the sequences of CD23 orthologs in a wide variety of mammals reveals that, although the residues that form the sugar-binding site in cow CD23 are present in most mammalian species, one or more of these residues are absent in many primate species (Fig. 12). In most of these cases, there are multiple mutations that would be expected to disrupt the Ca^2+^- and sugar-binding sites, although the specific mutations vary between different groups of primates and even between species within one group. The pattern of amino acid substitutions suggests that a mutation that resulted in loss of sugar-binding activity occurred in the progenitor to the branch of primates that includes the old and new world monkeys and the apes, but is separate from the lineage leading to tarsiers. Once sugar-binding activity was lost, selective pressure to retain other residues that form the Ca^2+^- and sugar-binding sites was absent, and additional mutations in key residues occurred.

## Discussion

**Figure 13.**
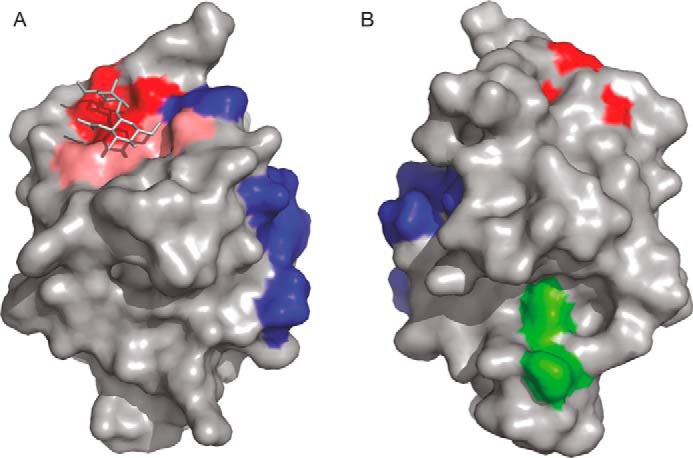
**Multiple binding interfaces of CD23.** Two views of the cow CD23 structure highlighting residues that interact with various ligands. *A*, view of the CRD with the disaccharide binding site exposed. *B*, view of the opposite face of the CRD. *Red*, residues that bind to sugars. *Blue*, portions of human CD23 that bind to IgE. *Green*, integrin-binding regions of human CD23. The binding site for CD21 in human CD23 maps to a C-terminal extension.

Demonstration that cow and mouse CD23 bind to sugars highlights a new class of ligands for this multifunctional receptor. Binding by the Ca^2+^-coordination mechanism common to most C-type CRDs involves a distinct face of the CRD compared with other ligands that interact with this domain, such as IgE and integrins. The distribution of these binding surfaces is summarized in Fig. 13. The fact that many of the interactions of CD23, such as the binding of IgE and binding of short consensus repeat domains 1 and 2 of CD21, are sugar-independent (24), is consistent with the distinct locations of the sugar-binding site and the sites on the surface of CD23 that bind these other ligands. Spatial separation of the binding sites for sugar and for CD21 suggests that the effect of CD21 glycosylation on binding to CD23 may be indirect rather than through the sugar-binding site (25). If the cow protein bound to IgE in the manner found in the human CD23, conformational changes would have to occur in loops 1 and 4, or perhaps in the IgE, that would potentially alter the sugar-binding site. Indeed, a structure of human CD23 with Ca^2+^ (13) shows changes in these loops that result in direct contacts with IgE.

The sugar-binding activity of cow CD23 is consistent with the presence of all of the ligands for the conserved Ca^2+^ required for interaction with sugars. One feature of this binding site is the Glu-Pro-Asn motif, which wraps around the Ca^2+^ and is associated with binding to sugars with equatorial 3- and 4-OH groups, such as mannose, GlcNAc, and glucose. The relatively open binding site allows interaction with these monosaccharides in multiple contexts, although some types of oligosaccharide ligands appear to be favored as a result of a small number of interactions with sugars beyond the terminal residues.

Mouse CD23 binds to a similar spectrum of ligands, but with distinctly weaker affinity. The reasons for this weaker affinity are not completely clear. The solid-phase binding competition assays are conducted under conditions in which the reporter ligand, horseradish peroxidase, is far below saturation, so the *K_I_* values measured for the monosaccharides correspond closely to their *K_D_* values. Thus, the results suggest fundamentally weaker binding at the primary monosaccharide-binding site. This difference is seen despite the fact that the coordination ligands for the primary Ca^2+^ are completely conserved and no other side chains within 4 Å of the monosaccharide residue in the primary binding site differ between cow and mouse CD23. The swap of aspartic acid for asparagine at position 254 and the reverse swap at position 260 would also be expected to preserve the secondary Ca^2+^-binding site. It is possible that changes farther from the sugar-binding site result in conformational changes affecting the positions of conserved residues in the primary binding site. In the secondary binding site, Ser^277^, which forms a hydrogen bond with O6 of GlcNAc, is found in cow and mouse, but not human, CD23, and Met^263^, which contacts C4 of GlcNAc, is conserved in cow, mouse, and human CD23. However, Leu^265^ in cow CD23, which contacts the carbonyl oxygen on the GlcNAc acetamido group, is replaced by Arg in mouse and human CD23, and it is not clear whether an equivalent contact would be made. The difference between cow and mouse CD23 at this position might further reduce the affinity for oligosaccharide ligands, in line with the negative glycan array results.

In early studies, Ca^2+^-dependent binding of mouse CD23 to monosaccharide-derivatized beads was reported based on an indirect assay, but no quantitative analysis was undertaken (24). The monosaccharide-binding characteristics of mouse CD23 reported in the present work are consistent with more recent results suggesting that mouse CD23 interacts with α-mannans and β-glucans from fungi (14). The α-mannans from *Candida albicans* and other yeasts are decorated with terminal 1–2-linked sugars that have exposed 3- and 4-OH groups that would be able to interact with the binding site in CD23, consistent with the observed binding to *Candida albicans* cells as well as to the α-mannan from *Saccharomyces cerevisiae* (26). In the case of *Aspergillus fumigatus*, the wall β-glucans contain appended mannose and GlcNAc residues that would be potential ligands (27). However, the fact that bacterial curdlan can be used to mimic the effects of the fungus suggests that glucose residues also can mediate binding to CD23 (14, 28). Although the main chain β1–3 linkages would block the 3-OH groups on glucose and prevent binding, glucose residues at the nonreducing termini of chains would be able to interact with CD23. Previous studies have suggested that human CD23 mediates pathogen recognition indirectly by forming complexes with IgE bound to mycobacteria or leishmania parasites (29, 30), but the results presented here and in the recent study of binding to fungi are consistent with a direct mechanism for mouse CD23 interacting with sugars on the surfaces of microorganisms. In cows, there might be a particular need for such direct recognition of microorganisms. As ruminants, cows may have high levels of nonpathogenic bacteria and fungi in the intestinal tract, and appropriate recognition may be necessary to maintain T cell anergy on the intestinal surfaces.

Absence of detectable sugar-binding activity for human CD23 might be expected, because one of the ligands at the conserved Ca^2+^ site is absent. Earlier results suggesting galactose-mediated binding of human CD23 to various glycoproteins are difficult to interpret, because the CD23 was not purified. It is therefore not clear whether CD23 itself was responsible for the interactions observed (31, 32). It is important to note that although the Glu-Pro-Asn sequence in the C-type CRDs in pulmonary surfactant protein A from various species is changed to either Glu-Pro-Ala or Glu-Pro-Arg, these CRDs still bind sugars, albeit with relatively weak affinity (33). However, it is clear from the results with mouse CD23 that factors in addition to the residues immediately around the conserved Ca^2+^ site contribute to forming a functional sugar-binding site, so a combination of changes can further reduce binding to the point where it is difficult or impossible to detect. It is striking that multiple additional changes at the Ca^2+^-binding sites occur in many of the primate species, which strongly suggests that the sugar-binding activity is not selected for in this evolutionary branch of the mammals.

Many of the glycan-binding receptors that have C-type CRDs are found on myeloid cells. The expression of CD23 on B cells as well is thus somewhat exceptional. The only other glycan-binding receptor with a C-type CRD found on B cells is prolectin (34). Comparison of the C-type CRDs reveals that the CRD of CD23 is more similar in sequence to prolectin than it is to any other CRD (8). In cows, the genes for prolectin and CD23 are both on chromosome 7, and in humans the genes for prolectin and CD23 are both located on chromosome 19. The gene for prolectin is absent from mice and other rodents but is present in humans and thus could possibly assume functions on B cells that human CD23 does not perform because of the loss of sugar-binding activity.

A key feature of the sugar-binding activity is sensitivity to pH. Loss of sugar-binding activity would occur in the pH range found in endosomal compartments and is consistent with a role for CD23 as a recycling endocytic receptor, which would bind ligands at the cell surface and release them following internalization, so that the receptor can return to the cell surface while the ligand is routed toward lysosomes for degradation. This function would depend on the presence of the Y*XX*L adapter protein–binding site in the cytoplasmic domain, which allows interaction with clathrin and is found in one of the two main splice variants of CD23 (5). The fact that this activity is present in CD23 expressed in some cells but not in others is consistent with the idea that CD23 plays multiple, distinct roles in different circumstances.

## Experimental procedures

### Cloning of expression constructs

A spleen cDNA library from *B. taurus* was obtained from AMS Biotechnology (Abingdon, UK), and a mouse spleen cDNA library was obtained from Takara Bio Europe (Saint-Germain-en-Laye, France). PCRs were conducted with Advantage 2 DNA polymerase (Takara). A preliminary heating step for 2 min at 95 °C was followed by 40 cycles of 30-s denaturation at 95 °C followed by 2-min renaturation and elongation at 65 °C. Fragments were isolated by agarose gel electrophoresis and either used for a second round of amplification or cloned into vector pCRII Topo using a TOPO cloning kit from Invitrogen (Paisley, UK). PCR primers used to amplify cow and mouse cDNAs, summarized in Figs. S2 and S3, were purchased from Invitrogen. For attachment of biotin tags, the 3′ primer included a sequence encoding the biotinylation sequence Leu-Asn-Asp-Ile-Phe-Glu-Ala-Gln-Lys-Ile-Glu-Trp-His-Glu appended to the C terminus of the protein. The sequence of a cDNA used for expression of the cow CRD without a biotin tag, synthesized by Invitrogen, is given in Fig. S4.

### Protein expression

All cloned fragments were inserted into expression vector pT5T (35) in *Escherichia coli* strain BL21(DE3) and grown in Luria-Bertani medium in the presence of 50 μg/ml ampicillin. For proteins with biotin tags, the bacteria also contained plasmid pBirA, which encodes biotin ligase (36), and cells were grown in the presence of both 50 μg/ml ampicillin and 50 μg/ml chloramphenicol. Overnight cultures (200 ml) grown at 25 °C were used to inoculate 6 liters of medium at 37 °C. Protein expression was induced with 100 μg/ml isopropyl-β-d-thiogalactoside when the *A*_550_ reached 0.7. For proteins with biotin tags, biotin was also added to a concentration of 12.5 μg/ml at the time of induction. After a further 2.5 h at 37 °C, cells were harvested by centrifugation.

Inclusion bodies were isolated by extensive sonication in 200 ml of 10 mm Tris-Cl, pH 7.8, followed by centrifugation at 15,000 × *g* for 15 min. The insoluble protein was dissolved in 100 ml of 6 m guanidine HCl, 100 mm Tris-Cl, pH 7.0, by homogenization. Fresh 2-mercaptoethanol was added to a final concentration of 0.01% and incubated at 4 °C for 30 min followed by centrifugation at 100,000 × *g* for 30 min. For renaturation of the cow CRD, the supernatant was dialyzed against three changes of 4 liters of 0.5 m NaCl, 25 mm Tris-Cl, pH 7.8, 25 mm CaCl_2_. The guanidine-solubilized mouse CRD was diluted into 400 ml of buffer before dialysis. After dialysis, insoluble protein was removed by centrifugation at 50,000 × *g* for 30 min.

Mannose-Sepharose affinity resin was prepared by divinyl sulfone coupling (37). Renatured proteins were applied to 10-ml columns, which were washed with 12 ml of 150 mm NaCl, 25 mm Tris-Cl, pH 7.8, 25 mm CaCl_2_ and eluted with 15 1-ml aliquots of 150 mm NaCl, 25 mm Tris-Cl, pH 7.8, 2.5 mm EDTA. Fractions containing protein were identified by analyzing aliquots by SDS-PAGE.

### Streptavidin complex formation

Biotin-tagged proteins were incubated overnight at 4 °C with fluorescently labeled streptavidin (Invitrogen) or streptavidin-alkaline phosphatase conjugate (Sigma) at a ratio of ∼2 mol of CRD to 1 mol of streptavidin monomer: 200 μg/ml CRD and 100 μg/ml streptavidin. Complexes of cow CRD were isolated by gel filtration on a Superdex S200 column, 1 × 30 cm, eluted at 0.5 ml/min with 100 mm NaCl, 10 mm Tris-Cl, pH 7.8, 2.5 mm EDTA. Complexes of mouse protein were repurified on 1-ml columns of mannose-Sepharose, which were washed with 1 ml of 150 mm NaCl, 25 mm Tris-Cl, pH 7.8, 25 mm CaCl_2_ and eluted with five 0.25-ml aliquots of 150 mm NaCl, 25 mm Tris-Cl, pH 7.8, 2.5 mm EDTA.

### Solid-phase binding assays

Biotin-tagged proteins, diluted to a concentration of ∼10 μg/ml in binding buffer (150 mm NaCl, 25 mm Tris-Cl, pH 7.8, 2.5 mm CaCl_2_) containing 0.1% BSA (Cohn Fraction V, Sigma), were applied to streptavidin-coated 96-well assay plates (Pierce). After incubation overnight at 4 °C with 100 μl of this coating solution, the wells were washed three times with binding buffer. Competing sugars were added to individual wells in binding buffer containing 0.1% BSA in the presence of horseradish peroxidase (Sigma). The concentration of horseradish peroxidase was 0.1 μg/ml for cow CD23 assays and 25 μg/ml for mouse CD23 assays. After incubation for 2 h at 4 °C, wells were washed five times with binding buffer and incubated with 3,3′,5,5′-tetramethyl benzidine peroxidase substrate (BioLegend). After color development for 5–30 min at room temperature, the reactions were stopped by the addition of an equal volume of 100 mm H_2_SO_4_, and the absorbance was read at 450 nm in a Victor3 plate reader (PerkinElmer Life Sciences).

Disaccharides and oligosaccharides for competition assays and crystallization were obtained from Dextra Laboratories, except that the Man_9_GlcNAc_2_ oligosaccharide from soybean agglutinin was isolated as described previously (38). For determination of pH dependence, Tris in the binding buffer was replaced with a mixture of 25 mm MES and 25 mm MOPS adjusted to appropriate pH values.

### Glycan array

Oligosaccharide arrays immobilized on glass slides were from the MicroArray Core Facility of the Scripps Research Institute (La Jolla, CA). The microarrays were composed of 600 defined glycans from the Consortium for Functional Glycomics printed on NHS-activated microarray slides using a contact printer in replicates of six as described previously (39). Absence of binding of uncomplexed streptavidin was checked on each batch as part of quality control with plant lectins (39). Labeled proteins were diluted in 150 mm NaCl, 20 mm Tris-Cl, pH 7.4, 2 mm CaCl_2_, 2 mm MgCl_2_, 0.05% Tween 20, 1% BSA and incubated with the slides. Slides were washed with 150 mm NaCl, 20 mm Tris-Cl, pH 7.4, 2 mm CaCl_2_, 2 mm MgCl_2_ and scanned with an InnoScan 1100AL scanner with data processed using Mapix 8.2.5 software (Innopsys, Chicago, IL). For each set of six replicate spots, the mean and S.D. were calculated after the highest and lowest values were excluded. Glycan microarray analyses were carried out according to the guidelines proposed by the MIRAGE initiative (40).

### Glycoprotein blotting experiments

Glycoprotein resolved by SDS-PAGE were blotted onto nitrocellulose membranes, which were blocked for 30 min at room temperature with 5% BSA in binding buffer as defined above for solid-phase binding assays. CRD-streptavidin-alkaline phosphatase complexes were added to a final concentration of 0.1–0.2 μg/ml and incubated for 60 min at room temperature. Following four 5-min washes with binding buffer, blots were developed with nitro blue tetrazolium/5-bromo-4-chloro-3-indolyl phosphate phosphatase substrate (Merck).

### Crystallization, data collection, and structure determination

Crystals of CD23 complexed with α-methyl mannoside were grown by hanging drop vapor diffusion at 16.5 °C using a mixture of 0.9:0.9 μl of protein/reservoir solution in each drop, with the protein solution comprising 4.2 mg/ml protein, 5 mm CaCl_2_, 10 mm Tris-Cl, pH 8.0, 25 mm NaCl, and 50 mm α-methyl mannoside. The reservoir solution contained 16% PEG 4000, 0.1 m MES, pH 5.5. Crystals were dipped in a freezing solution containing 30% PEG 4000, 0.1 m MES, pH 5.5, 25 mm NaCl, 5 mm CaCl_2_, and 50 mm α-methyl mannoside before being frozen in liquid nitrogen for data collection. Crystals of CD23 with GlcNAcβ1–2Man were grown at 22 °C using a mixture of 0.9:1.8 μl of protein/reservoir solution, with the protein solution containing 5 mg/ml protein, 5 mm CaCl_2_, 10 mm Tris-Cl, pH 8.0, 25 mm NaCl, and 50 mm GlcNAcβ1–2Man. The reservoir solution contained 24% PEG 8000, 0.1 m HEPES, pH 7.0. Crystals were frozen directly from the drop in liquid nitrogen for data collection. Crystals of CD23 complexed with GlcNAc_2_Man_3_ were grown at 16.5 °C using a mixture of 0.9:0.9 μl of protein/reservoir solution. The protein solution comprised 5 mg/ml protein, 5 mm CaCl_2_, 10 mm Tris-Cl, pH 8.0, 25 mm NaCl, and 10 mm GlcNAc_2_Man_3_. The reservoir solution contained 22% PEG 8000, 0.1 m HEPES, pH 7.0. Crystals were frozen directly from the drop in liquid nitrogen for data collection.

Diffraction data were measured at 100 K on Beamline 12-2 at the Stanford Synchrotron Radiation Laboratory. Diffraction data were integrated with XDS and scaled with AIMLESS (41) to a maximum resolution of 1.0 Å for the complex with α-methyl mannoside, 1.2 Å for the complex with GlcNAcβ1–2Man, and 2.7 Å for the complex with GlcNAc_2_Man_3_. Data statistics are summarized in Table 1.

**Table 1:** **Crystallographic data statistics**

Parameters	CD23 α-methyl mannoside complex	CD23 GlcNAcβ1–2Man complex	CD23 GlcNAc_2_Man_3_ complex
Symmetry	P2_1_2_1_2_1_	P2_1_2_1_2_1_	P3_1_21
Wavelength (Å)	0.81566	0.97946	0.97946
Unit cell lengths (Å)	*a* = 38.55, *b* = 45.92, *c* = 69.27	*a* = 37.41, *b* = 45.71, *c* = 69.11	*a* = *b* = 33.17, *c* = 207.81
Resolution Å	1.00 (1.02–1.00)*^a^*	1.20 (1.22–1.20)	2.7 (2.83)
*R*_sym_*^b^*	3.3 (11.9)	6.6 (15.2)	10.5 (34.4)
Mn(I) half-set correlation CC(1/2)	0.998 (0.990)	0.993 (0.977)	0.996 (0.985)
Mean((*I*)/σ(*I*))	33.5 (12.3)	16.9 (9.5)	13.7 (3.1)
Percentage completeness	99.7 (98.5)	99.7 (98.9)	99.5 (96.9)
No. of unique reflections	67,049	37,766	4156
Average multiplicity	6.4 (5.8)	6.3 (5.6)	16.9 (12.9)

*^a^* Values shown in parentheses are for the last shell.

*^b^ R*_sym_ = 100 × ∑*_h_*∑*_I_* (|*I_i_*(*h*) − 〈*I*(*h*)〉|)/∑*_h_*∑*_i_ I_i_*(*h*), where *Ii*(*h*) = observed intensity, and 〈*I*(*h*)〉 = mean intensity obtained from multiple measurements.

The structure of CD23 complexed with α-methyl mannoside was solved by molecular replacement with Phaser (42). The model used for molecular replacement was prepared from the coordinates of the human CD23 structure, Protein Data Bank entry 2H2T, using the protein chain and the Ca^2+^ ion. The molecular replacement solution confirmed that the space group was P2_1_2_1_2_1_, with one CD23 molecule in the asymmetric unit. Difference Fourier maps revealed that in addition to the primary Ca^2+^, there is an additional Ca^2+^ ion and that the α-methyl mannoside is bound to the primary Ca^2+^ ion in two orientations.

The crystals of CD23 complexed with GlcNAcβ1–2Man had the same symmetry and unit cell parameters as the complex with α-methyl mannoside, so the structure was solved by rigid body refinement starting with the CRD from the model of the α-methyl mannoside complex, with two Ca^2+^ ions and with one water molecule bound to the secondary Ca^2+^ ion. The carbohydrate and other water molecules were removed from the initial model. The same *R*_free_ reflections were chosen for both complexes.

The structure of CD23 complexed with GlcNAc_2_Man_3_ was solved by molecular replacement using the same initial model used for the CD23 GlcNAcβ1–2Man complex. The molecular replacement solution confirmed that the space group was P3_1_21, with one CD23 molecule in the asymmetric unit. Difference Fourier maps revealed that the GlcNAc_2_Man_3_ oligosaccharide cross-links two symmetry-related CD23 molecules. The sugar, which can be described as a central Man residue with two GlcNAcβ1–2Man moieties linked to the 3- and 6-OH groups, sits on a 2-fold axis. The two GlcNAcβ1–2Man groups of the carbohydrate superimpose closely on one another and are well-defined in the electron density maps. The mannose moiety of each GlcNAcβ1–2Man branch binds the primary Ca^2+^ in one unit cell and the primary Ca^2+^ in a symmetry-related unit cell. The central mannose residue of the pentasaccharide has two conformations, each at 50% occupancy, with the GlcNAcβ1–2Man moiety from either the α1–3- or α1–6-linked branch bound to the protein. Because the carbohydrate is on a 2-fold axis, it is defined with 0.5 occupancy, and the nonbonded interactions for the carbohydrate with symmetry-related carbohydrate were turned off.

Model building and refinement were performed with Coot and PHENIX (43, 44). Refinement included individual positional and isotropic temperature factor refinement of the sugar and water molecules and the protein of the GlcNAcβ1–2Man and GlcNAc_2_Man_3_ complex. Anisotropic temperature factor refinement was used for the protein in the high-resolution α-methyl mannoside complex. For the α-methyl mannoside complex, riding hydrogen atoms were added to the protein and the carbohydrate. TLS refinement, secondary structure restraints, and reference model restraints were used in the refinement of the GlcNAc_2_Man_3_ complex. Refinement statistics are shown in Table 2.

**Table 2:** **Crystallographic refinement statistics**

Parameters	CD23 α-methyl mannoside complex	CD23 GlcNAcβ1–2Man complex	CD23 GlcNAc_2_Man_3_ complex
No. of reflections used for refinement	63,632 [[[R-89]]]	35,787 [[[R-23]]]	3926 [[[R-19]]]
Reflections marked for *R*_free_	3350	1890	183
*R*_free_*^a^*	12.8	16.5	25.1
*R*_cryst_*^a^*	11.4	15.1	19.9
Average *B* factor (Å^2^)	10.2	10.1	85.3
Bond length root mean square deviation (Å)	0.006	0.006	0.012
Angle root mean square deviation (degrees)	1.04	0.92	1.46
Ramachandran plot: (percentage in each region):*^b^* preferred/allowed/outliers	94.3/5.7/0.0	95.1/4.9/0.0	93.7/6.3/0.0
PDB codes	6PWS	6PWR	6PWT

*^a^R* and *R*_free_ = 100 × ∑*_h_*|F*_o_*(*h*) − *F_c_*(*h*)|/∑*_h_F_o_*(*h*), where *F_o_*(*h*) = observed structure factor amplitude and *F_c_*(*h*) = calculated structure factor amplitude for the working and test sets, respectively.

*^b^* As defined in Coot.

### Flow cytometry

Peripheral blood mononuclear cells were isolated by density gradient centrifugation (Lymphoprep, STEMCELL Technologies, Cambridge, UK), using whole blood from cattle sampled under home office license (PPL7009059), and then stored in liquid nitrogen. Prior to staining, the cells were defrosted and rested for 2 h at 37 °C with 5% CO_2_ in RPMI 1640 medium (Gibco, Thermo Fisher Scientific, Hemel Hempstead, UK) supplemented with 10% fetal bovine serum (Sigma-Aldrich, Poole, UK). The following antibodies were used to stain the cells in this study: CD23 (9p25, IgG1, Dendritics, Lyon, France), CD21 conjugates with R-phycoerythrin (CC51, IgG2b, Bio-Rad Laboratories, Watford, UK), IgG1 isotype control (15H6, SouthernBiotech, Birmingham, AL), IgG2b conjugates with R-phycoerythrin isotype control (Bio-Rad Laboratories), and rabbit F(ab′)_2_ anti-mouse IgG conjugates with FITC (Bio-Rad Laboratories). Staining with each antibody was performed in PBS containing 2% BSA for 30 min at 4 °C. Flow cytometry data were acquired on a FACSCalibur (BD Biosciences, Reading, UK), and analysis was performed using FlowJo version 10 (FlowJo, LLC, Ashland, OR).

### Sequence identification and alignment

CD23 sequences were identified in the National Center for Biotechnology Information database and were aligned manually and arranged by animal phylogeny (45).

## Author contributions

S. A. F. J., D. F. S., D. W., K. D., W. I. W., and M. E. T. conceptualization; S. A. F. J., H. F., L. J., Y. L., D. F. S., D. W., K. D., W. I. W., and M. E. T. formal analysis; S. A. F. J., Y. L., D. F. S., D. W., K. D., W. I. W., and M. E. T. supervision; S. A. F. J., Y. L., D. F. S., D. W., K. D., W. I. W., and M. E. T. funding acquisition; S. A. F. J., H. F., A. G. M., A. H., A. M., Z. H., L. J., Y. L., D. F. S., D. W., K. D., W. I. W., and M. E. T. investigation; S. A. F. J., H. F., D. W., K. D., W. I. W., and M. E. T. visualization; S. A. F. J., H. F., A. G. M., D. W., K. D., W. I. W., and M. E. T. writing-original draft; S. A. F. J., Y. L., D. F. S., D. W., K. D., W. I. W., and M. E. T. project administration; S. A. F. J., H. F., A. G. M., A. H., A. M., Z. H., L. J., Y. L., D. F. S., D. W., K. D., W. I. W., and M. E. T. writing-review and editing.

## Supplementary Material

Supporting Information

## Footnotes

4 A. Holder and D. Werling, unpublished data.

## Footnotes

3 The abbreviations used are:

CRD: carbohydrate-recognition domain
PDB: Protein Data Bank.
