# Supplementary material for: CD23 is a glycan-binding receptor in some mammalian species
Source: J Biol Chem. 2019 Sep 5;294(41):14845–59. doi: 10.1074/jbc.RA119.010572 (PMC6791321; doi:10.1074/jbc.RA119.010572)
Supplement: Supporting Information [file supp_294_41_14845__index.html]

CD23 is a glycan-binding receptor in some mammalian species — Sugar-binding activity of CD23 — CD23 is a glycan-binding receptor in some mammalian species — EDITORS' PICK: Sugar-binding activity of CD23 — Supporting Information 

# CD23 is a glycan-binding receptor in some mammalian species

## Supporting Information

- Supporting Information (to be published online) - Figures S1-S4 Table S1
