# Supplementary material for: CD23 is a glycan-binding receptor in some mammalian species
Source: J Biol Chem. 2019 Sep 5;294(41):14845–59. doi: 10.1074/jbc.RA119.010572 (PMC6791321; doi:10.1074/jbc.RA119.010572)
Supplement: Supporting Information [file supp_RA119.010572_155092_1_supp_387680_px2333.pdf]

Supporting Information

CD23 is a glycan-binding receptor in some mammalian species

Sabine A. F. Jégouzo, Hadar Feinberg, Andrew Morrison, Angela Holder, Alisha May, Zhiyao Huang, Linghua Jiang, Yi Lasanajak, David F. Smith, Dirk Werling, Kurt Drickamer, William I. Weis, and Maureen E. Taylor

Table S1. Glycan array results for cow CD23 binding to the Consortium for Functional Glycomics array version 5.2.

Figure S1. Sequences of C-type lectin-like domains encoded in CD23 genes in mammals.

Figure S2. PCR primers for cow CD23 cDNA.

Figure S3. PCR primers for mouse CD23 cDNA.

Figure S4. Sequence of codon-optimized cDNA for the CRD from cow CD23 without biotin tag.

Table S1

Cow CD23 binding to array of synthetic glycans from the Consortium for Functional Glycomics

Strepatividin-CRD complex was incubated with the array at a concentration of 100 μg/ml.

Glycan number	Glycan structure	Average	Standard deviation
470	GlcNAcβ1-6(GlcNAcβ1-2)Manα1-6(GlcNAcβ1-2Manα1-3)Manβ1-4GlcNAcβ1-4(Fucα1-6)GlcNAcb-Sp24	4720	449
417	GlcNAcβ1-2(GlcNAcβ1-6)Manα1-6(GlcNAcβ1-2Manα1-3)Manβ1-4GlcNAcβ1-4GlcNAcb-Sp19	1818	285
321	Galβ1-3GlcNAcβ1-2Manα1-6(Galβ1-3GlcNAcβ1-2Manα1-3)Manβ1-4GlcNAcβ1-4GlcNAcb-Sp19	1743	228
53	GlcNAcβ1-2Manα1-6(GlcNAcβ1-2Manα1-3)Manβ1-4GlcNAcβ1-4GlcNAcb-Sp13	1568	103
468	Fucα1-2Galβ1-4(Fucα1-3)GlcNAcβ1-2Manα1-6(Fucα1-2Galβ1-4(Fucα1-3)GlcNAcβ1-2Manα1-3)Manβ1-4GlcNAcβ1-4(Fucα1-6)GlcNAcb-Sp24	1551	70
351	Galβ1-3GlcNAcβ1-2Manα1-6(Galβ1-3GlcNAcβ1-2Manα1-3)Manβ1-4GlcNAcβ1-4(Fucα1-6)GlcNAcb-Sp22	1369	313
52	GlcNAcβ1-2Manα1-6(GlcNAcβ1-2Manα1-3)Manβ1-4GlcNAcβ1-4GlcNAcb-Sp12	1329	175
521	Neu5Acα2-3Galβ1-3GlcNAcβ1-2Mana-Sp0	1269	236
214	Manα1-2Manα1-2Manα1-6(Manα1-3)Mana-Sp9	1247	127
306	Neu5Acα2-6Galβ1-4GlcNAcβ1-2Manα1-6(GlcNAcβ1-2Manα1-3)Manβ1-4GlcNAcβ1-4GlcNAcb-Sp12	1148	316
427	GlcNAcβ1-2Manα1-6(GlcNAcβ1-4)(GlcNAcβ1-2Manα1-3)Manβ1-4GlcNAcβ1-4GlcNAc-Sp21	1120	457
596	Neu5Acα2-6Galβ1-4GlcNAcβ1-3Galβ1-4GlcNAcβ1-3Galβ1-4GlcNAcβ1-2Manα1-6(Neu5Acα2-6Galβ1-4GlcNAcβ1-3Galβ1-4GlcNAcβ1-3Galβ1-4GlcNAcβ1-2Manα1-3)Manβ1-4GlcNAcβ1-4GlcNAcb-Sp12	1117	93
424	Galβ1-3GlcNAcβ1-6(Galβ1-3GlcNAcβ1-2)Manα1-6(Galβ1-3GlcNAcβ1-2Manα1-3)Manβ1-4GlcNAcβ1-4GlcNAcb-Sp19	1044	190
349	GlcNAcβ1-2Manα1-6(GlcNAcβ1-2Manα1-3)Manβ1-4GlcNAcβ1-4(Fucα1-6)GlcNAcb-Sp22	886	247
108	Galα1-3(Fucα1-2)Galb-Sp18	864	71
314	Manα1-2Manα1-6(Manα1-2Manα1-3)Manα1-6(Manα1-2Manα1-2Manα1-3)Mana-Sp9	856	51
536	Fucα1-2Galβ1-4GlcNAcβ1-3Galβ1-4GlcNAcβ1-2Manα1-6(Fucα1-2Galβ1-4GlcNAcβ1-3Galβ1-4GlcNAcβ1-2Manα1-3)Manβ1-4GlcNAcβ1-4GlcNAcb-Sp24	850	28
358	Fucα1-2Galβ1-4GlcNAcβ1-2Manα1-6(Fucα1-2Galβ1-4GlcNAcβ1-2Manα1-3)Manβ1-4GlcNAcβ1-4GlcNAcb-Sp20	826	111
215	Manα1-6(Manα1-3)Manα1-6(Manα1-2Manα1-3)Manβ1-4GlcNAcβ1-4GlcNAcb-Sp12	815	27
522	Galα1-3Galβ1-3GlcNAcβ1-2Mana-Sp0	784	114
72	Fucα1-2Galβ1-4(Fucα1-3)GlcNAcb-Sp8	783	157
525	GlcNAcβ1-2 Manα1-6(GlcNAcβ1-4)(GlcNAcβ1-2Manα1-3)Manβ1-4GlcNAcβ1-4(Fucα1-6)GlcNAc-Sp21	770	209
518	GalNAcα1-3(Fucα1-2)Galβ1-4 GlcNAcβ1-2Mana-Sp0	769	119
76	Fucα1-2Galβ1-4GlcNAcb-Sp8	743	118
89	GalNAcα1-3(Fucα1-2)Galb-Sp8	730	53
519	Galβ1-3GlcNAcβ1-2Mana-Sp0	723	46
394	Galβ1-4GlcNAcβ1-2Manα1-6(GlcNAcβ1-2Manα1-3)Manβ1-4GlcNAcβ1-4GlcNAc-Sp12	690	112
90	GalNAcα1-3(Fucα1-2)Galb-Sp18	662	33
405	Galα1-3(Fucα1-2)Galβ1-4(Fucα1-3)Glcb-Sp21	646	81
539	Galβ1-4GlcNAcβ1-3Galβ1-4GlcNAcβ1-3Galβ1-4GlcNAcβ1-2Manα1-6(Galβ1-4GlcNAcβ1-3Galβ1-4GlcNAcβ1-3Galβ1-4GlcNAcβ1-2Manα1-3)Manβ1-4GlcNAcβ1-4GlcNAcb-Sp12	627	120
202	GlcAb-Sp8	623	49
313	Manα1-2Manα1-6(Manα1-3)Manα1-6(Manα1-2Manα1-2Manα1-3)Mana-Sp9	608	89
71	Fucα1-2Galβ1-4(Fucα1-3)GlcNAcb-Sp0	595	84
412	Fucα1-2Galβ1-4(Fucα1-3)GlcNAcβ1-3GalNAca-Sp14	477	26
368	Galα1-3(Fucα1-2)Galβ1-4GlcNAcβ1-2Manα1-6(Galα1-3(Fucα1-2)Galβ1-4GlcNAcβ1-2Manα1-3)Manβ1-4GlcNAcβ1-4GlcNAcb-Sp20	472	124
367	GalNAcα1-3(Fucα1-2)Galβ1-4GlcNAcβ1-2Manα1-6(GalNAcα1-3(Fucα1-2)Galβ1-4GlcNAcβ1-2Manα1-3)Manβ1-4GlcNAcβ1-4GlcNAcb-Sp20	468	51
480	Galβ1-4GlcNAcβ1-6(Galβ1-4GlcNAcβ1-2)Manα1-6(Galβ1-4GlcNAcβ1-2Manα1-3)Manβ1-4GlcNAcβ1-4(Fucα1-6)GlcNAcb-Sp24	465	28
414	GalNAcα1-3(Fucα1-2)Galβ1-4(Fucα1-3)GlcNAcβ1-3GalNAc-Sp14	456	27
238	Neu5Acα2-3Galβ1-3(Fucα1-4)GlcNAcb-Sp8	419	13
297	Neu5Acα2-3Galβ1-4(Fucα1-3)GlcNAcβ1-6(Galβ1-3)GalNAca-Sp14	405	58
393	GlcNAcβ1-2Manα1-6(Galβ1-4GlcNAcβ1-2Manα1-3)Manβ1-4GlcNAcβ1-4GlcNAc-Sp12	405	115
548	GlcNAcβ1-3Galβ1-4GlcNAcβ1-6(GlcNAcβ1-3Galβ1-4GlcNAcβ1-2)Manα1-6(GlcNAcβ1-3Galβ1-4GlcNAcβ1-2Man α1-3)Manβ1-4GlcNAcβ1-4GlcNAc-Sp24	396	83
104	Galα1-3(Fucα1-2)Galβ1-4(Fucα1-3)GlcNAcb-Sp8	364	48

561	Galβ1-3GlcNAcβ1-3Galβ1-4GlcNAcβ1-3Galβ1-4GlcNAcβ1-6(Galβ1-3GlcNAcβ1-3Galβ1-4GlcNAcβ1-3Galβ1-4GlcNAβ1-2)Manα1-6(Galβ1-3GlcNAcβ1-3Galβ1-4GlcNAcβ1-3Galβ1-4GlcNAcβ1-2Manα1-3)Manβ1-4GlcNAcβ1-4(Fucα1-6)GlcNAcb-Sp24	358	101
208	Manα1-2Manα1-6(Manα1-2Manα1-3)Mana-Sp9	356	51
207	Manα1-2Manα1-2Manα1-3Mana-Sp9	317	166
359	Fucα1-2Galβ1-4(Fucα1-3)GlcNAcβ1-2Manα1-6(Fucα1-2Galβ1-4(Fucα1-3)GlcNAcβ1-2Manα1-3)Manβ1-4GlcNAcβ1-4GlcNAb-Sp20	312	13
533	Galβ1-3GalNAcβ1-3Gal-Sp21	311	46
362	Fucα1-4(Galβ1-3)GlcNAcβ1-2Manα1-6(Fucα1-4(Galβ1-3)GlcNAcβ1-2Manα1-3)Manβ1-4GlcNAcβ1-4(Fucα1-6)GlcNAcb-Sp22	307	37
28	(3S)Galβ1-3(Fucα1-4)GlcNAcb-Sp8	297	55
429	GlcNAcβ1-6(GlcNAcβ1-2)Manα1-6(GlcNAcβ1-4)(GlcNAcβ1-2Manα1-3)Manβ1-4GlcNAcβ1-4GlcNAc-Sp21	294	76
70	Fucα1-2Galβ1-4(Fucα1-3)GlcNAcβ1-3Galβ1-4(Fucα1-3)GlcNAcβ1-3Galβ1-4(Fucα1-3)GlcNAcb-Sp0	291	53
24	(3S)Galβ1-4(Fucα1-3)(6S)Glc-Sp0	272	18
572	Galβ1-4GlcNAcβ1-3Galβ1-4GlcNAcβ1-6(Galβ1-4GlcNAcβ1-3Galβ1-4GlcNAβ1-2)Manα1-6(Galβ1-4GlcNAcβ1-3Galβ1-4GlcNAcβ1-2Manα1-3)Manβ1-4GlcNAcβ1-4(Fucα1-6)GlcNAcb-Sp24	258	31
464	Galα1-3(Fucα1-2)Galβ1-3GalNAcb-Sp8	241	34
597	Neu5Acα2-3Galβ1-4GlcNAcβ1-3Galβ1-4GlcNAcβ1-3Galβ1-4GlcNAcβ1-2Manα1-6(Neu5Acα2-3Galβ1-4GlcNAcβ1-3Galβ1-4GlcNAcβ1-3Galβ1-4GlcNAcβ1-2Manα1-3)Manβ1-4GlcNAcβ1-4GlcNAcb-Sp12	233	42
248	Neu5Acα2-3Galβ1-3GlcNAcb-Sp0	227	17
83	GalNAcα1-3(Fucα1-2)Galβ1-4(Fucα1-3)GlcNAcb-Sp0	226	13
562	Galβ1-3GlcNAcβ1-3Galβ1-4GlcNAcβ1-6(Galβ1-3GlcNAcβ1-3Galβ1-4GlcNAβ1-2)Manα1-6(Galβ1-3GlcNAcβ1-3Galβ1-4GlcNAcβ1-2Manα1-3)Manβ1-4GlcNAcβ1-4(Fucα1-6)GlcNAcb-Sp24	225	57
445	Fucα1-2Galβ1-4GlcNAcβ1-6(Fucα1-2Galβ1-4GlcNAcβ1-3)GalNAc-Sp14	225	74
471	Galβ1-3GlcNAcβ1-2Manα1-6(GlcNAcβ1-4)(Galβ1-3GlcNAcβ1-2Manα1-3)Manβ1-4GlcNAcβ1-4GlcNAcb-Sp21	216	104
237	Neu5Acα2-3Galβ1-3(6S)GlcNAc-Sp8	199	52
350	Galβ1-4GlcNAcβ1-2Manα1-6(Galβ1-4GlcNAcβ1-2Manα1-3)Manβ1-4GlcNAcβ1-4(Fucα1-6)GlcNAcb-Sp22	193	9
534	GlcNAcβ1-3Galβ1-4GlcNAcβ1-2Manα1-6(GlcNAcβ1-3Galβ1-4GlcNAcβ1-2Manα1-3)Manβ1-4GlcNAcβ1-4GlcNAcb-Sp12	187	41
420	GalNAcα1-3(Fucα1-2)Galβ1-3GlcNAcβ1-3GalNAc-Sp14	183	49
334	Neu5Acα2-3Galβ1-4(Fucα1-3)GlcNAcβ1-6(Neu5Acα2-3Galβ1-3)GalNAc-Sp14	182	30
374	Neu5Acα2-3Galβ1-4(Fucα1-3)GlcNAcβ1-3GalNAca-Sp14	180	23
75	Fucα1-2Galβ1-4GlcNAcb-Sp0	179	55
88	GlcNAcβ1-3Galβ1-3GalNAca-Sp8	179	16
442	Fucα1-2Galβ1-4(Fucα1-3)GlcNAcβ1-2Manα1-6(Fucα1-2Galβ1-4(Fucα1-3)GlcNAcβ1-4(Fucα1-2Galβ1-4(Fucα1-3)GlcNAcβ1-2)Manα1-3)Manβ1-4GlcNAcβ1-4GlcNAcb-Sp12	178	37
289	Galβ1-4(Fucα1-3)(6S)Glcb-Sp0	169	25
450	GalNAcα1-3(Fucα1-2)Galβ1-4GlcNAcβ1-2Manα1-6(GalNAcα1-3(Fucα1-2)Galβ1-4GlcNAcβ1-2Manα1-3)Manβ1-4GlcNAcβ1-4(Fucα1-6)GlcNAcb-Sp22	168	56
103	Galα1-3(Fucα1-2)Galβ1-4(Fucα1-3)GlcNAcb-Sp0	166	32
566	GlcNAcβ1-3Galβ1-4GlcNAcβ1-3Galβ1-4GlcNAcβ1-2Manα1-6(GlcNAcβ1-3Galβ1-4GlcNAcβ1-3Galβ1-4GlcNAcβ1-2Manα1-3)Manβ1-4GlcNAcβ1-4(Fucα1-6)GlcNAcb-Sp24	165	68
97	GalNAcβ1-4(Fucα1-3)GlcNAcb-Sp0	164	18
356	KDNα2-3Galβ1-3GalNAca-Sp14	162	55
255	Neu5Acα2-3Galβ1-4(Fucα1-3)GlcNAcβ1-3Galβ1-4GlcNAcb-Sp8	161	24
397	Galβ1-4(Fucα1-3)GlcNAcβ1-3GalNAca-Sp14	159	53
119	Galα1-4(Fucα1-2)Galβ1-4GlcNAcb-Sp8	151	18
466	Glcα1-4Glcα1-4Glcα1-4Glcb-Sp10	150	22
179	GlcNAcβ1-3GalNAca-Sp8	150	13
278	Neu5Gcα2-3Galβ1-3(Fucα1-4)GlcNAcb-Sp0	147	24
415	Galβ1-4(Fucα1-3)GlcNAcβ1-2Manα1-6(Galβ1-4(Fucα1-3)GlcNAcβ1-2Manα1-3)Manβ1-4GlcNAcβ1-4(Fucα1-6)GlcNAcb-Sp22	146	26
413	Galα1-3(Fucα1-2)Galβ1-4(Fucα1-3)GlcNAcβ1-3GalNAc-Sp14	144	95
537	GlcNAcβ1-3Galβ1-4GlcNAcβ1-3Galβ1-4GlcNAcβ1-2Manα1-6(GlcNAcβ1-3Galβ1-4GlcNAcβ1-3Galβ1-4GlcNAcβ1-2Manα1-3)Manβ1-4GlcNAcβ1-4GlcNAcb-Sp12	142	10
423	Galα1-3(Fucα1-2)Galβ1-4GlcNAcβ1-2Manα1-6(Galα1-3(Fucα1-2)Galβ1-4GlcNAcβ1-2Manα1-3)Manβ1-4GlcNAcβ1-4(Fucα1-6)GlcNAcb-Sp22	142	42
598	Neu5Acα2-6Galβ1-4GlcNAcβ1-3Galβ1-4GlcNAcβ1-2Manα1-6(Neu5Acα2-6Galβ1-4GlcNAcβ1-3Galβ1-4GlcNAcβ1-2Manα1-3)Manβ1-4GlcNAcβ1-4GlcNAcb-Sp12	142	23
555	Galβ1-4GlcNAcβ1-3Galβ1-4GlcNAcβ1-3Galβ1-4GlcNAcβ1-3Galβ1-4GlcNAcβ1-3Galβ1-4GlcNAcβ1-2Manα1-6(Galβ1-4GlcNAcβ1-3Galβ1-4GlcNAcβ1-3Galβ1-4GlcNAcβ1-3Galβ1-4GlcNAcβ1-3Galβ1-4GlcNAcβ1-2Manα1-3)Manβ1-4GlcNAcβ1-4GlcNAcb-Sp25	142	30
251	Neu5Acα2-3Galβ1-4(Fucα1-3)GlcNAcβ1-3Galβ1-4(Fucα1-3)GlcNAcβ1-3Galβ1-4(Fucα1-3)GlcNAcb-Sp0	141	23
290	Galβ1-4(Fucα1-3)GlcNAcβ1-3Galβ1-3(Fucα1-4)GlcNAcb-Sp0	140	19

357	Fucα1-2Galβ1-3GlcNAcβ1-2Manα1-6(Fucα1-2Galβ1-3GlcNAcβ1-2Manα1-3)Manβ1-4GlcNAcβ1-4GlcNAcb-Sp20	140	10
400	Galα1-4Galβ1-4GlcNAcβ1-2Manα1-6(Galα1-4Galβ1-4GlcNAcβ1-2Manα1-3)Manβ1-4GlcNAcβ1-4GlcNAcb-Sp24	138	47
416	Fucα1-2Galβ1-4GlcNAcβ1-2Manα1-6(Fucα1-2Galβ1-4GlcNAcβ1-2Manα1-3)Manβ1-4GlcNAcβ1-4(Fucα1-6)GlcNAcb-Sp22	138	3
132	Galβ1-4GlcNAcβ1-6GalNAc-Sp14	137	31
487	(3S)Galβ1-3(Fucα1-4)GlcNAcb-Sp0	137	54
443	Galβ1-4(Fucα1-3)GlcNAcβ1-6GalNAc-Sp14	133	8
192	GlcNAcβ1-6GalNAca-Sp8	132	17
465	Glcα1-6Glcα1-6Glcα1-6Glcb-Sp10	131	28
477	Neu5Acα2-6Galβ1-4GlcNAcβ1-2Manα1-6(Neu5Acα2-6Galβ1-4GlcNAcβ1-2Manα1-3)Manβ1-4GlcNAcβ1-4(Fucα1-6)GlcNAcb-Sp24	131	25
247	Fucα1-2(6S)Galβ1-4Glcb-Sp0	131	22
528	Fucα1-4(Galβ1-3)GlcNAcβ1-2 Mana-Sp0	131	31
496	Fucα1-2(6S)Galβ1-3(6S)GlcNAcb-Sp0	129	41
260	Fucα1-2Galβ1-4(6S)Glcb-Sp0	128	39
288	Galβ1-4(Fucα1-3)(6S)GlcNAcb-Sp0	127	8
448	Neu5Acα2-8Neu5Acα2-3Galβ1-3GalNAcβ1-4(Neu5Acα2-8Neu5Acα2-3)Galβ1-4Glcb-Sp0	125	32
219	(3S)Galβ1-4(Fucα1-3)(6S)GlcNAcb-Sp8	124	19
451	Galα1-3(Fucα1-2)Galβ1-3GlcNAcβ1-2Manα1-6(Galα1-3(Fucα1-2)Galβ1-3GlcNAcβ1-2Manα1-3)Manβ1-4GlcNAcβ1-4(Fucα1-6)GlcNAcb-Sp22	120	18
193	GlcNAcβ1-6GalNAca-Sp14	118	22
390	Galα1-3Galβ1-3GlcNAcβ1-2Manα1-6(Galα1-3Galβ1-3GlcNAcβ1-2Manα1-3)Manβ1-4GlcNAcβ1-4GlcNAc-Sp19	118	51
80	Fucα1-4GlcNAcb-Sp8	118	35
593	Neu5Acα2-3Galβ1-4GlcNAcβ1-3Galβ1-4GlcNAcβ1-2Manα1-6(Neu5Acα2-3Galβ1-4GlcNAcβ1-3Galβ1-4GlcNAcβ1-2Manα1-3)Manβ1-4GlcNAcβ1-4GlcNAcb-Sp12	116	26
312	Manα1-6(Manα1-3)Manα1-6(Manα1-3)Manb-Sp10	115	10
245	Neu5Acα2-3Galβ1-3GalNAcβ1-3Galα1-4Galβ1-4Glcb-Sp0	114	34
328	Neu5Acα2-3Galβ1-3(Fucα1-4)GlcNAcβ1-3Galβ1-3(Fucα1-4)GlcNAcb-Sp0	113	15
216	Manα1-6(Manα1-3)Manα1-6(Manα1-3)Manβ1-4GlcNAcβ1-4GlcNAcb-Sp12	112	9
126	Galβ1-3(Fucα1-4)GlcNAcβ1-3Galβ1-4(Fucα1-3)GlcNAcb-Sp0	112	14
127	Galβ1-3GlcNAcβ1-3Galβ1-4(Fucα1-3)GlcNAcb-Sp0	112	21
355	KDNα2-3Galβ1-4Glc-Sp0	112	19
369	Galα1-3Galβ1-4(Fucα1-3)GlcNAcβ1-2Manα1-6(Galα1-3Galβ1-4(Fucα1-3)GlcNAcβ1-2Manα1-3)Manβ1-4GlcNAcβ1-4GlcNAcb-Sp20	111	5
403	Galβ1-3GlcNAcα1-6Galβ1-4GlcNAcb-Sp0	111	52
168	Galβ1-4GlcNAcb-Sp0	111	24
489	Fucα1-2Galβ1-4GlcNAcβ1-6GalNAca-Sp14	109	21
145	Galβ1-3GalNAcβ1-4Galβ1-4Glcb-Sp8	109	13
261	Neu5Acα2-3Galβ1-4Glcb-Sp0	109	17
391	Galα1-3Galβ1-3(Fucα1-4)GlcNAcβ1-2Manα1-6(Galα1-3Galβ1-3(Fucα1-4)GlcNAcβ1-2Manα1-3)Manβ1-4GlcNAcβ1-4GlcNAc-Sp19	108	16
388	GalNAcβ1-4(Neu5Acα2-3)Galβ1-4GlcNAcβ1-3GalNAca-Sp14	108	20
346	Galβ1-4GlcNAcβ1-2Manα1-3Manβ1-4GlcNAcβ1-4GlcNAc-Sp12	107	3
535	GlcNAcβ1-3Galβ1-4GlcNAcβ1-2Manα1-6(GlcNAcβ1-3Galβ1-4GlcNAcβ1-2Manα1-3)Manβ1-4GlcNAcβ1-4GlcNAcb-Sp25	107	17
352	(6S)GlcNAcβ1-3Galβ1-4GlcNAcb-Sp0	105	24
195	Glcα1-4Glcb-Sp8	105	22
375	GalNAcβ1-4GlcNAcβ1-2Manα1-6(GalNAcβ1-4GlcNAcβ1-2Manα1-3)Manβ1-4GlcNAcβ1-4GlcNAc-Sp12	105	20
79	Fucα1-3GlcNAcb-Sp8	104	17
111	Galα1-3GalNAca-Sp16	103	6
371	Fucα1-4(Fucα1-2Galβ1-3)GlcNAcβ1-2Manα1-3(Fucα1-4(Fucα1-2Galβ1-3)GlcNAcβ1-2Manα1-3)Manβ1-4GlcNAcβ1-4GlcNAcb-Sp19	103	28
453	GalNAcα1-3(Fucα1-2)Galβ1-3GlcNAcβ1-2Manα1-6(GalNAcα1-3(Fucα1-2)Galβ1-3GlcNAcβ1-2Manα1-3)Manβ1-4GlcNAcβ1-4(Fucα1-6)GlcNAcb-Sp22	103	8
32	(3S)Galβ1-4(Fucα1-3)GlcNAc-Sp0	103	7
446	Galα1-3(Fucα1-2)Galβ1-4GlcNAcβ1-6(Galα1-3(Fucα1-2)Galβ1-4GlcNAcβ1-3)GalNAc-Sp14	103	31
554	GlcNAcβ1-3Galβ1-4GlcNAcβ1-3Galβ1-4GlcNAcβ1-3Galβ1-4GlcNAcβ1-2Manα1-6(GlcNAcβ1-3Galβ1-4GlcNAcβ1-3Galβ1-4GlcNAcβ1-3Galβ1-4GlcNAcβ1-3Galβ1-4GlcNAcβ1-2Manα1-3)Manβ1-4GlcNAcβ1-4GlcNAcb-Sp25	102	32
568	GlcNAcβ1-3Galβ1-4GlcNAcβ1-3Galβ1-4GlcNAcβ1-3Galβ1-4GlcNAcβ1-2Manα1-6(GlcNAcβ1-3Galβ1-4GlcNAcβ1-3Galβ1-4GlcNAcβ1-3Galβ1-4GlcNAcβ1-2Manα1-3)Manβ1-4GlcNAcβ1-4(Fucα1-6)GlcNAcb-Sp24	102	50
353	KDNα2-3Galβ1-4(Fucα1-3)GlcNAc-Sp0	102	12

84	(3S)Galβ1-4(Fucα1-3)Glcb-Sp0	101	19
272	Neu5Acα2-6Galb-Sp8	100	16
120	Galα1-4Galβ1-4GlcNAcb-Sp0	99	11
320	Neu5Gcb2-6Galβ1-4GlcNAc-Sp8	99	7
165	Galβ1-4GlcNAcβ1-3Galβ1-4Glcb-Sp8	98	34
268	Neu5Acα2-6Galβ1-4GlcNAcβ1-3Galβ1-4(Fucα1-3)GlcNAcβ1-3Galβ1-4(Fucα1-3)GlcNAcb-Sp0	97	8
161	Galβ1-4GlcNAcβ1-3Galβ1-4(Fucα1-3)GlcNAcβ1-3Galβ1-4(Fucα1-3)GlcNAcb-Sp0	96	14
581	Galβ1-4GlcNAcβ1-3Galβ1-4GlcNAcβ1-6(Galβ1-4GlcNAcβ1-3Galβ1-4GlcNAcβ1-3)GalNAca-Sp14	95	5
318	Neu5Acα2-8Neu5Acb-Sp17	95	9
218	Neu5Acα2-3Galβ1-4GlcNAcβ1-3Galβ1-4(Fucα1-3)GlcNAcb-Sp0	95	12
330	Galα1-4Galβ1-4GlcNAcβ1-3Galβ1-4Glcb-Sp0	94	25
454	Galβ1-4GlcNAcβ1-6(Galβ1-4GlcNAcβ1-2)Manα1-6(Galβ1-4GlcNAcβ1-2Manα1-3)Manβ1-4GlcNAcβ1-4GlcNAcb-Sp19	94	7
54	Galβ1-4GlcNAcβ1-2Manα1-6(Galβ1-4GlcNAcβ1-2Manα1-3)Manβ1-4GlcNAcβ1-4GlcNAcb-Sp12	94	15
469	Fucα1-2Galβ1-3(Fucα1-4)GlcNAcβ1-2Manα1-6(Fucα1-2Galβ1-3(Fucα1-4)GlcNAcβ1-2Manα1-3)Manβ1-4GlcNAcβ1-4(Fucα1-6)GlcNAcβ1-4(Fucα1-6)GlcNAcb-Sp19	94	18
167	Galβ1-4GlcNAcβ1-6(Galβ1-3)GalNAc-Sp14	93	9
236	Neu5Acα2-3GalNAcβ1-4GlcNAcb-Sp0	93	38
550	Galα1-3Galβ1-4GlcNAcβ1-2Manα1-6(Galα1-3Galβ1-4GlcNAcβ1-2Manα1-3)Manβ1-4GlcNAcβ1-4GlcNAc-Sp24	93	16
549	Galβ1-4GlcNAcβ1-3Galβ1-4GlcNAcβ1-6(Galβ1-4GlcNAcβ1-3Galβ1-4GlcNAcβ1-2)Manα1-6(Galβ1-4GlcNAcβ1-3Galβ1-4GlcNAcβ1-2Manα1-3)Manα1-4GlcNAcβ1-4GlcNAc-Sp24	92	32
143	Galβ1-3GalNAcβ1-3Galα1-4Galβ1-4Glcb-Sp0	92	6
240	Neu5Acα2-3Galβ1-4(Neu5Acα2-3Galβ1-3)GlcNAcb-Sp8	92	20
370	GalNAcα1-3(Fucα1-2)Galβ1-3GlcNAcβ1-2Manα1-6(GalNAcα1-3(Fucα1-2)Galβ1-3GlcNAcβ1-2Manα1-3)Manβ1-4GlcNAcβ1-4GlcNAcb-Sp20	92	25
360	Galα1-3Galβ1-4GlcNAcβ1-2Manα1-6(Galα1-3Galβ1-4GlcNAcβ1-2Manα1-3)Manβ1-4GlcNAcβ1-4GlcNAcb-Sp20	91	12
384	GlcNAcβ1-2Manα1-6(GlcNAcβ1-4(GlcNAcβ1-2)Manα1-3)Manβ1-4GlcNAcβ1-4GlcNAc-Sp21	91	14
380	Galβ1-4GlcNAcβ1-6(Fucα1-4(Fucα1-2Galβ1-3)GlcNAcβ1-3)Galβ1-4Glc-Sp21	91	17
93	GalNAcα1-4(Fucα1-2)Galβ1-4GlcNAcb-Sp8	91	9
166	Galβ1-4GlcNAcβ1-6(Galβ1-3)GalNAca-Sp8	90	15
441	Fucα1-2Galβ1-4 GlcNAcβ1-2Manα1-6(Fucα1-2Galβ1-4GlcNAcβ1-2(Fucα1-2Galβ1-4GlcNAcβ1-4)Manα1-3)Manβ1-4GlcNAcβ1-4GlcNAcb-Sp12	90	14
96	GalNAcβ1-3Galα1-4Galβ1-4GlcNAcb-Sp0	90	10
204	GlcAβ1-6Galb-Sp8	90	19
339	GlcNAcα1-4Galβ1-4GlcNAcβ1-3Galβ1-4(Fucα1-3)GlcNAcβ1-3Galβ1-4(Fucα1-3)GlcNAcb-Sp0	89	8
213	Manα1-6(Manα1-3)Mana-Sp9	89	6
78	Fucα1-2Galb-Sp8	89	19
100	Galα1-2Galb-Sp8	88	34
107	Galα1-3(Fucα1-2)Galb-Sp8	88	17
222	Fucα1-2(6S)Galβ1-4(6S)Glcb-Sp0	88	2
300	Galβ1-4GlcNAcβ1-6(Galβ1-4GlcNAcβ1-3)Galβ1-4GlcNAc-Sp0	88	21
256	Neu5Acα2-3Galβ1-4GlcNAcβ1-3Galβ1-4GlcNAcβ1-3Galβ1-4GlcNAcb-Sp0	88	11
449	GalNAcβ1-4Galβ1-4Glcb-Sp0	88	28
131	Galβ1-4GlcNAcβ1-6GalNAca-Sp8	87	20
541	Galβ1-3GlcNAcβ1-3Galβ1-4GlcNAcβ1-2Manα1-6(Galβ1-3GlcNAcβ1-3Galβ1-4GlcNAcβ1-2Manα1-3)Manβ1-4GlcNAcβ1-4GlcNAc-Sp25	87	10
283	Neu5Gcα2-6GalNAca-Sp0	87	21
478	Neu5Acα2-3Galβ1-4GlcNAcβ1-2Manα1-6(Neu5Acα2-3Galβ1-4GlcNAcβ1-2Manα1-3)Manβ1-4GlcNAcβ1-4(Fucα1-6)GlcNAcb-Sp24	87	20
354	KDNα2-6Galβ1-4GlcNAc-Sp0	87	38
467	Neu5Acα2-3Galβ1-4GlcNAcβ1-6(Neu5Acα2-3Galβ1-4GlcNAcβ1-3)GalNAca-Sp14	86	18
82	GalNAcα1-3(Fucα1-2)Galβ1-3GlcNAcb-Sp0	86	39
189	GlcNAcβ1-4GlcNAcβ1-4GlcNAcβ1-4GlcNAcβ1-4GlcNAcβ1-4GlcNAcβ1-Sp8	86	6
150	Galβ1-3GlcNAcb-Sp8	85	20
102	Galα1-3(Fucα1-2)Galβ1-3GlcNAcb-Sp8	85	22
162	Galβ1-4GlcNAcβ1-3Galβ1-4GlcNAcβ1-3Galβ1-4GlcNAcb-Sp0	85	12
128	Galβ1-3(Fucα1-4)GlcNAc-Sp0	84	13
239	Neu5Acα2-3Galβ1-3(Fucα1-4)GlcNAcβ1-3Galβ1-4(Fucα1-3)GlcNAcb-Sp0	84	23

571	Galβ1-4GlcNAcβ1-3Galβ1-4GlcNAcβ1-3Galβ1-4GlcNAcβ1-3Galβ1-4GlcNAcβ1-3Galβ1-4GlcNAcβ1-2Manα1-6(Galβ1-4GlcNAcβ1-3Galβ1-4GlcNAcβ1-3Galβ1-4GlcNAcβ1-3Galβ1-4GlcNAcβ1-3Galβ1-4GlcNAcβ1-2Manα1-3)Manβ1-4GlcNAcβ1-4(Fucα1-6)GlcNAcb-Sp19	84	52
510	(3S)GalNAcβ1-4GlcNAc-Sp8	84	7
512	Galβ1-4(6P)GlcNAcb-Sp0	84	17
121	Galα1-4Galβ1-4GlcNAcb-Sp8	84	28
284	Neu5Gcα2-6Galβ1-4GlcNAcb-Sp0	84	10
271	Neu5Acα2-6Galβ1-4Glcb-Sp8	83	11
331	GalNAcβ1-3Galα1-4Galβ1-4GlcNAcβ1-3Galβ1-4Glcb-Sp0	83	13
488	Galβ1-4(Fucα1-3)GlcNAcβ1-6(Neu5Acα2-6(Neu5Acα2-3Galβ1-3)GlcNAcβ1-3)Galβ1-4Glc-Sp21	83	6
153	Galβ1-4(Fucα1-3)GlcNAcβ1-3Galβ1-4(Fucα1-3)GlcNAcb-Sp0	83	12
57	Neu5Acα2-6Galβ1-4GlcNAcβ1-2Manα1-6(Neu5Acα2-6Galβ1-4GlcNAcβ1-2Manα1-3)Manβ1-4GlcNAcβ1-4GlcNAcb-Sp24	82	7
177	GlcNAcβ1-6(GlcNAcβ1-3)GalNAca-Sp14	82	24
304	GalNAcβ1-3Galb-Sp8	82	33
399	Galα1-4Galβ1-3GlcNAcβ1-2Manα1-6(Galα1-4Galβ1-3GlcNAcβ1-2Manα1-3)Manβ1-4GlcNAcβ1-4GlcNAcb-Sp19	82	7
473	Neu5Acα2-3Galβ1-4GlcNAcβ1-2Mana-Sp0	82	17
77	Fucα1-2Galβ1-4Glcb-Sp0	82	13
326	Neu5,9Ac2α2-3Galβ1-3GlcNAcb-Sp0	82	9
156	Galβ1-4(6S)Glcb-Sp8	81	27
199	Glcβ1-6Glcb-Sp8	81	16
378	Galβ1-3GlcNAcβ1-3Galβ1-4GlcNAcβ1-6(Galβ1-3GlcNAcβ1-3)Galβ1-4Glcb-Sp0	81	36
531	GalNAcα1-3(Fucα1-2)Galβ1-3GalNAcβ1-3Galα1-4Galβ1-4Glc-Sp21	81	8
69	Fucα1-2Galβ1-4(Fucα1-3)GlcNAcβ1-3Galβ1-4(Fucα1-3)GlcNAcb-Sp0	81	8
285	Neu5Gca-Sp8	81	22
500	Fucα1-2Galβ1-3GlcNAcβ1-6(Fucα1-2Galβ1-3GlcNAcβ1-3)GalNAca-Sp14	81	34
122	Galα1-4Galβ1-4Glcb-Sp0	80	19
295	(6S)Galβ1-4(6S)GlcNAcb-Sp0	80	25
419	Galα1-3(Fucα1-2)Galβ1-3GlcNAcβ1-3GalNAc-Sp14	80	7
553	GalNAcβ1-4GlcNAcβ1-3GalNAcβ1-4GlcNAcb-Sp0	80	18
585	GlcNAcβ1-3Galβ1-4GlcNAcβ1-6(GlcNAcβ1-3Galβ1-4GlcNAcβ1-3)GalNAca-Sp14	80	11
234	Neu5Acα2-6(Neu5Acα2-3)GalNAca-Sp8	79	27
299	Neu5Acα2-6Galβ1-4GlcNAcβ1-2Manα1-6(Galβ1-4GlcNAcβ1-2Manα1-3)Manβ1-4GlcNAcβ1-4GlcNAcb-Sp12	79	13
144	Galβ1-3GalNAcβ1-4(Neu5Acα2-3)Galβ1-4Glcb-Sp0	79	7
282	Neu5Gcα2-3Galβ1-4Glcb-Sp0	79	12
333	GalNAcα1-3(Fucα1-2)Galβ1-4GlcNAcβ1-3Galβ1-4GlcNAcβ1-3Galβ1-4GlcNAcb-Sp0	79	16
444	Galβ1-4GlcNAcβ1-2Mana-Sp0	79	18
133	GlcNAcβ1-6(Galβ1-3)GalNAca-Sp8	79	21
556	GlcNAβ1-3Galβ1-3GalNAc-Sp14	79	59
115	Galα1-3Galβ1-4GlcNAcb-Sp8	78	8
233	Neu5Acα2-3Galβ1-3GalNAcβ1-4(Neu5Acα2-3)Galβ1-4Glcb-Sp0	78	11
81	Fucβ1-3GlcNAcb-Sp8	77	7
366	Galβ1-4GlcNAcβ1-2Manα1-6(Galβ1-4GlcNAcβ1-4(Galβ1-4GlcNAcβ1-2)Manα1-3)Manβ1-4GlcNAcβ1-4GlcNAc-Sp21	77	15
142	Galβ1-3GalNAcb-Sp8	77	16
94	GalNAcβ1-3GalNAca-Sp8	77	6
95	GalNAcβ1-3(Fucα1-2)Galb-Sp8	77	11
34	(3S)Galβ1-4(6S)GlcNAcb-Sp0	77	5
178	GlcNAcβ1-6(GlcNAcβ1-3)Galβ1-4GlcNAcb-Sp8	77	15
262	Neu5Acα2-3Galβ1-4Glcb-Sp8	76	9
187	GlcNAcβ1-6(GlcNAcβ1-4)GalNAca-Sp8	76	10
101	Galα1-3(Fucα1-2)Galβ1-3GlcNAcb-Sp0	76	10
332	GalNAcα1-3(Fucα1-2)Galβ1-4GlcNAcβ1-3Galβ1-4GlcNAcb-Sp0	76	21
33	(3S)Galβ1-4(Fucα1-3)GlcNAc-Sp8	76	11
60	Fucα1-2Galβ1-3(Fucα1-4)GlcNAcb-Sp8	76	11

136	Neu5Acα2-6(Galβ1-3)GalNAca-Sp14	76	18
517	Galα1-3(Fucα1-2)Galβ1-4GlcNAcβ1-2Mana-Sp0	76	12
191	GlcNAcβ1-4GlcNAcβ1-4GlcNAcb-Sp8	75	10
20	Galβ1-4GlcNAcβ1-6(Galβ1-4GlcNAcβ1-3)GalNAc-Sp14	75	20
109	Galα1-4(Galα1-3)Galβ1-4GlcNAcb-Sp8	75	16
225	GalNAcβ1-4(Neu5Acα2-8Neu5Acα2-8Neu5Acα2-8Neu5Acα2-3)Galβ1-4Glcb-Sp0	75	15
509	GalNAcβ1-4(6S)GlcNAc-Sp8	75	16
50	Manα1-6(Manα1-3)Manβ1-4GlcNAcβ1-4GlcNAcb-Sp12	75	25
298	Galβ1-3Galβ1-4GlcNAcb-Sp8	75	11
205	KDNα2-3Galβ1-3GlcNAcb-Sp0	74	17
565	Galβ1-4GlcNAcβ1-3Galβ1-4GlcNAcβ1-2Manα1-6(Galβ1-4GlcNAcβ1-3Galβ1-4GlcNAcβ1-2Manα1-3)Manβ1-4GlcNAcβ1-4(Fucα1-6)GlcNAcb-Sp24	74	9
226	GalNAcβ1-4(Neu5Acα2-8Neu5Acα2-8Neu5Acα2-3)Galβ1-4Glcb-Sp0	74	13
229	Neu5Acα2-8Neu5Acα2-8Neu5Aca-Sp8	74	18
303	Galβ1-4GlcNAcβ1-6Galβ1-4GlcNAcb-Sp0	74	13
463	Galα1-3(Fucα1-2)Galβ1-3GalNAca-Sp8	74	6
486	Neu5Acα2-3Galβ1-3GlcNAcβ1-6GalNAca-Sp14	74	9
243	Neu5Acα2-6(Neu5Acα2-3Galβ1-3)GalNAca-Sp14	74	19
327	Neu5Acα2-6Galβ1-4GlcNAcβ1-3Galβ1-3GlcNAcb-Sp0	74	12
311	Manα1-6Manb-Sp10	73	10
110	Galα1-3GalNAca-Sp8	73	16
365	Galβ1-4(Fucα1-3)GlcNAcβ1-6(Fucα1-2Galβ1-4GlcNAcβ1-3)Galβ1-4Glc-Sp21	73	8
379	Galβ1-4(Fucα1-3)GlcNAcβ1-6(Galβ1-3GlcNAcβ1-3)Galβ1-4Glc-Sp21	73	10
294	4S(3S)Galβ1-4GlcNAcb-Sp0	72	6
599	GlcNAcβ1-3Fuca-Sp21	72	5
257	Neu5Acα2-3Galβ1-4GlcNAcb-Sp0	72	5
141	Galβ1-3GalNAca-Sp16	72	2
56	Neu5Acα2-6Galβ1-4GlcNAcβ1-2Manα1-6(Neu5Acα2-6Galβ1-4GlcNAcβ1-2Man-α1-3)Manβ1-4GlcNAcβ1-4GlcNAcb-Sp21	71	14
402	Galβ1-3GlcNAcβ1-6Galβ1-4GlcNAcb-Sp0	71	8
582	Neu5Acα2-3Galβ1-4GlcNAcβ1-3Galβ1-4GlcNAcβ1-3GalNAca-Sp14	71	15
105	Galα1-3(Fucα1-2)Galβ1-4GlcNAc-Sp0	71	8
172	Galβ1-4Glcb-Sp8	71	5
196	Glcα1-4Glca-Sp8	71	18
249	Neu5Acα2-3Galβ1-4(6S)GlcNAcb-Sp8	70	11
188	GlcNAcβ1-4Galβ1-4GlcNAcb-Sp8	70	8
287	Galβ1-3GlcNAcβ1-3Galβ1-3GlcNAcb-Sp0	70	9
47	(6S)GlcNAcb-Sp8	70	12
523	GalNAcβ1-4GlcNAcβ1-2Mana-Sp0	70	10
551	GlcNAcβ1-3Galβ1-4GlcNAcβ1-6(GlcNAcβ1-3Galβ1-3)GalNAca-Sp14	70	23
230	GalNAcβ1-4(Neu5Acα2-3)Galβ1-4GlcNAcb-Sp0	69	19
426	Fucα1-3GlcNAcβ1-6(Galβ1-4GlcNAcβ1-3)Galβ1-4Glc-Sp21	69	16
345	Neu5Acα2-6Galβ1-4GlcNAcβ1-2Manα1-3Manβ1-4GlcNAcβ1-4GlcNAc-Sp12	69	17
485	Galβ1-3(Fucα1-4)GlcNAcβ1-6GalNAca-Sp14	69	24
563	Neu5Acα2-8Neu5Acα2-3Galβ1-3GalNAcβ1-4(Neu5Acα2-3)Galβ1-4Glc-Sp21	69	19
67	Fucα1-2Galβ1-3GlcNAcb-Sp0	69	12
235	Neu5Acα2-3GalNAca-Sp8	69	9
170	Galβ1-4GlcNAcb-Sp23	68	8
308	GlcNAcβ1-4GlcNAcb-Sp10	68	33
335	GlcNAcα1-4Galβ1-4GlcNAcβ1-3Galβ1-4GlcNAcβ1-3Galβ1-4GlcNAcb-Sp0	68	12
462	Neu5Acα2-6Galβ1-4GlcNAcβ1-6(Neu5Acα2-6Galβ1-4GlcNAcβ1-2)Manα1-6(GlcNAcβ1-4)(Neu5Acα2-6Galβ1-4GlcNAcβ1-4(Neu5Acα2-6Galβ1-4GlcNAcβ1-2)Manα1-3)Manβ1-4GlcNAcβ1-4GlcNAcb-Sp21	68	7
11	Neu5Acb-Sp8	68	20
123	Galα1-4GlcNAcb-Sp8	68	4

35	(3S)Galβ1-4(6S)GlcNAcb-Sp8	68	16
270	Neu5Acα2-6Galβ1-4Glcb-Sp0	68	8
286	Neu5Acα2-3Galβ1-4GlcNAcβ1-6(Galβ1-3)GalNAca-Sp14	68	8
340	GlcNAcα1-4Galβ1-4GlcNAcβ1-3Galβ1-4GlcNAcb-Sp0	68	14
408	Neu5Acα2-3Galβ1-3GalNAcβ1-4(Neu5Acα2-8Neu5Acα2-3)Galβ1-4Glcb-Sp0	68	13
154	Galβ1-4(Fucα1-3)GlcNAcβ1-3Galβ1-4(Fucα1-3)GlcNAcβ1-3Galβ1-4(Fucα1-3)GlcNAcb-Sp0	67	11
220	Fucα1-2(6S)Galβ1-4GlcNAcb-Sp0	67	11
157	Galβ1-4GalNAcα1-3(Fucα1-2)Galβ1-4GlcNAcb-Sp8	67	13
258	Neu5Acα2-3Galβ1-4GlcNAcb-Sp8	67	11
337	GlcNAcα1-4Galβ1-3GlcNAcb-Sp0	67	20
203	GlcAβ1-3Galb-Sp8	66	7
307	GlcNAcβ1-3Man-Sp10	66	24
495	Fucα1-2Galβ1-3(6S)GlcNAcb-Sp0	66	8
155	Galβ1-4(6S)Glcb-Sp0	66	3
253	Neu5Acα2-3Galβ1-4(Fucα1-3)GlcNAcb-Sp8	66	17
151	Galβ1-4(Fucα1-3)GlcNAcb-Sp0	66	14
263	Neu5Acα2-6GalNAca-Sp8	66	39
323	Neu5Acα2-3Galβ1-4GlcNAcβ1-2Manα1-6(Neu5Acα2-6Galβ1-4GlcNAcβ1-2Manα1-3)Manβ1-4GlcNAcβ1-4GlcNAcb-Sp12	66	24
580	Galβ1-4GlcNAcβ1-3Galβ1-4GlcNAcβ1-6(Galβ1-3)GalNAca-Sp14	66	21
447	GalNAcα1-3(Fucα1-2)Galβ1-4GlcNAcβ1-6(GalNAcα1-3(Fucα1-2)Galβ1-4GlcNAcβ1-3)GalNAc-Sp14	65	15
532	Galα1-3(Fucα1-2)Galβ1-3GalNAcβ1-3Galα1-4Galβ1-4Glc-Sp21	65	13
64	Fucα1-2Galβ1-3GalNAcβ1-4(Neu5Acα2-3)Galβ1-4Glcb-Sp9	65	5
273	Neu5Acα2-8Neu5Aca-Sp8	65	8
422	Fucα1-2Galβ1-3GlcNAcβ1-2Manα1-6(Fucα1-2Galβ1-3GlcNAcβ1-2Manα1-3)Manβ1-4GlcNAcβ1-4(Fucα1-6)GlcNAcb-Sp22	65	10
482	Neu5Acα2-6Galβ1-4GlcNAcβ1-6(Fucα1-2Galβ1-4(Fucα1-3)GlcNAcβ1-3)Galβ1-4Glc-Sp21	65	13
529	Neu5Acα2-3Galβ1-4(Fucα1-3)GlcNAcβ1-2Mana-Sp0	65	12
159	Galβ1-4GlcNAcβ1-3GalNAca-Sp8	65	13
217	Manβ1-4GlcNAcb-Sp0	64	17
63	Fucα1-2Galβ1-3GalNAcβ1-4(Neu5Acα2-3)Galβ1-4Glcb-Sp0	64	10
212	Manα1-2Manα1-6(Manα1-2Manα1-3)Manα1-6(Manα1-2Manα1-2Manα1-3)Manβ1-4GlcNAcβ1-4GlcNAcb-Sp12	64	3
325	Neu5,9Ac2α2-3Galβ1-4GlcNAcb-Sp0	64	44
176	GlcNAcβ1-6(GlcNAcβ1-3)GalNAca-Sp8	64	7
197	Glcα1-6Glcα1-6Glcb-Sp8	64	31
206	KDNα2-3Galβ1-4GlcNAcb-Sp0	64	13
250	Neu5Acα2-3Galβ1-4(Fucα1-3)(6S)GlcNAcb-Sp8	64	8
301	GlcNAcβ1-6(Galβ1-4GlcNAcβ1-3)Galβ1-4GlcNAc-Sp0	64	5
310	MurNAcβ1-4GlcNAcb-Sp10	64	7
584	GlcNAcβ1-3Galβ1-4GlcNAcβ1-6(Galβ1-3)GalNAca-Sp14	64	15
266	Neu5Acα2-6Galβ1-4GlcNAcb-Sp0	63	18
267	Neu5Acα2-6Galβ1-4GlcNAcb-Sp8	63	14
269	Neu5Acα2-6Galβ1-4GlcNAcβ1-3Galβ1-4GlcNAcb-Sp0	63	5
116	Galα1-3Galβ1-4Glcb-Sp0	63	14
293	Neu5Acα2-3Galβ1-4GlcNAcβ1-3Galβ1-3GlcNAcb-Sp0	63	4
396	Fucα1-2Galβ1-4GlcNAcβ1-3GalNAca-Sp14	63	16
23	6S(3S)Galβ1-4GlcNAcb-Sp0	62	7
130	Fucα1-4(Galβ1-3)GlcNAcb-Sp8	62	10
474	Neu5Acα2-3Galβ1-4GlcNAcβ1-6GalNAca-Sp14	62	9
65	Fucα1-2Galβ1-3GlcNAcβ1-3Galβ1-4Glcb-Sp8	62	9
329	Neu5Acα2-6Galβ1-4GlcNAcβ1-3Galβ1-4GlcNAcβ1-3Galβ1-4GlcNAcb-Sp0	62	7
600	Galβ1-3GalNAcβ1-4(Neu5Acα2-8Neu5Acα2-8Neu5Acα2-3)Galβ1-4Glcb-Sp21	62	9
259	Neu5Acα2-3Galβ1-4GlcNAcβ1-3Galβ1-4GlcNAcb-Sp0	62	6
524	Neu5Acα2-3Galβ1-3GalNAcβ1-4Galβ1-4Glcb-Sp0	62	13

227	Neu5Acα2-8Neu5Acα2-8Neu5Acα2-3Galβ1-4Glcb-Sp0	62	5
343	Manα1-6(Neu5Acα2-6Galβ1-4GlcNAcβ1-2Manα1-3)Manβ1-4GlcNAcβ1-4GlcNAc-Sp12	62	9
385	Fucα1-2Galβ1-3GalNAcα1-3(Fucα1-2)Galβ1-4Glcb-Sp0	62	9
7	Fuca-Sp9	61	19
13	Glcb-Sp8	61	2
91	GalNAcα1-3GalNAcb-Sp8	61	12
305	GlcAβ1-3GlcNAcb-Sp8	61	12
149	Galβ1-3GlcNAcb-Sp0	61	23
99	GalNAcβ1-4GlcNAcb-Sp8	61	5
254	Neu5Acα2-3Galβ1-4(Fucα1-3)GlcNAcβ1-3Galb-Sp8	61	8
296	(6P)Glcb-Sp10	61	8
430	GlcNAcβ1-6(GlcNAcβ1-2)Manα1-6(GlcNAcβ1-4)(GlcNAcβ1-4(GlcNAcβ1-2)Manα1-3)Manβ1-4GlcNAcβ1-4GlcNAc-Sp21	61	7
55	Neu5Acα2-6Galβ1-4GlcNAcβ1-2Manα1-6(Neu5Acα2-6Galβ1-4GlcNAcβ1-2Manα1-3)Manβ1-4GlcNAcβ1-4GlcNAcb-Sp12	61	7
175	GlcNAcβ1-2Galβ1-3GalNAca-Sp8	61	13
124	Galα1-6Glcb-Sp8	60	7
586	Neu5Acα2-3Galβ1-4GlcNAcβ1-3Galβ1-4GlcNAcβ1-6(Neu5Acα2-3Galβ1-4GlcNAcβ1-3Galβ1-4GlcNAcβ1-3)GalNAca-Sp14	60	9
182	GlcNAcβ1-3Galβ1-4GlcNAcb-Sp0	60	15
194	GlcNAcβ1-6Galβ1-4GlcNAcb-Sp8	60	14
418	Fucα1-2Galβ1-3GlcNAcβ1-3GalNAc-Sp14	60	17
398	GalNAcα1-3GalNAcβ1-3Galα1-4Galβ1-4GlcNAcb-Sp0	60	24
579	Galβ1-4GlcNAcβ1-3Galβ1-4GlcNAcβ1-3GalNAca-Sp14	60	30
395	Neu5Acα2-3Galβ1-3GlcNAcβ1-3GalNAca-Sp14	60	19
434	Galβ1-4GlcNAcβ1-6(Galβ1-4GlcNAcβ1-2)Manα1-6(GlcNAcβ1-4)(Galβ1-4GlcNAcβ1-4(Galβ1-4GlcNAcβ1-2)Manα1-3)Manβ1-4GlcNAcβ1-4GlcNAc-Sp21	60	3
377	Galβ1-3GalNAcα1-3(Fucα1-2)Galβ1-4GlcNAc-Sp0	59	20
386	Fucα1-2Galβ1-3GalNAcα1-3(Fucα1-2)Galβ1-4GlcNAcb-Sp0	59	11
401	Galα1-3Galβ1-4GlcNAcβ1-3GalNAca-Sp14	59	19
476	Neu5Acα2-6Galβ1-4 GlcNAcβ1-6(Neu5Acα2-6Galβ1-4GlcNAcβ1-3)GalNAca-Sp14	59	11
73	Fucα1-2Galβ1-4GlcNAcβ1-3Galβ1-4GlcNAcb-Sp0	59	13
174	GlcNAcα1-6Galβ1-4GlcNAcb-Sp8	59	7
590	Neu5Acα2-3Galβ1-4GlcNAcβ1-3Galβ1-4GlcNAcβ1-6(Galβ1-3)GalNAca-Sp14	59	8
322	Neu5Acα2-3Galβ1-4GlcNAcβ1-2Manα1-6(Neu5Acα2-3Galβ1-4GlcNAcβ1-2Manα1-3)Manβ1-4GlcNAcβ1-4GlcNAcb-Sp12	59	17
342	Neu5Acα2-6Galβ1-4GlcNAcβ1-2Manα1-6(Manα1-3)Manβ1-4GlcNAcβ1-4GlcNAc-Sp12	59	4
309	GlcNAcβ1-4GlcNAcb-Sp12	59	5
148	Galβ1-3GlcNAcβ1-3Galβ1-4Glcb-Sp10	58	9
36	(3S)Galβ1-4GlcNAcb-Sp0	58	20
336	GlcNAcα1-4Galβ1-4GlcNAcb-Sp0	58	10
475	Neu5Acα2-6Galβ1-4GlcNAcβ1-6GalNAca-Sp14	58	16
40	(4S)Galβ1-4GlcNAcb-Sp8	58	15
244	Neu5Acα2-3Galb-Sp8	58	4
31	(3S)Galβ1-3GlcNAcb-Sp8	57	12
373	Neu5Acα2-6Galβ1-4GlcNAcβ1-3GalNAc-Sp14	57	18
381	Galβ1-4(Fucα1-3)GlcNAcβ1-6(Fucα1-4(Fucα1-2Galβ1-3)GlcNAcβ1-3)Galβ1-4Glc-Sp21	57	8
211	Manα1-2Manα1-6(Manα1-3)Manα1-6(Manα1-2Manα1-2Manα1-3)Manβ1-4GlcNAcβ1-4GlcNAcb-Sp12	57	17
452	Neu5Acα2-6Galβ1-4GlcNAcβ1-6(Fucα1-2Galβ1-3GlcNAcβ1-3)Galβ1-4Glc-Sp21	57	4
180	GlcNAcβ1-3GalNAca-Sp14	57	7
200	G-ol-Sp8	57	4
439	(6S)Galβ1-3GlcNAcb-Sp0	57	5
98	GalNAcβ1-4GlcNAcb-Sp0	57	17
209	Manα1-2Manα1-3Mana-Sp9	57	5
292	Neu5Acα2-3Galβ1-3GlcNAcβ1-3Galβ1-3GlcNAcb-Sp0	57	11
498	GalNAcβ1-4(Fucα1-3)(6S)GlcNAcb-Sp8	57	13
185	GlcNAcβ1-3Galβ1-4Glcb-Sp0	56	13

228	GalNAcβ1-4(Neu5Acα2-8Neu5Acα2-3)Galβ1-4Glcb-Sp0	56	14
280	Neu5Gcα2-3Galβ1-4(Fucα1-3)GlcNAcb-Sp0	56	13
324	Galβ1-4(Fucα1-3)GlcNAcβ1-2Manα1-6(Galβ1-4(Fucα1-3)GlcNAcβ1-2Manα1-3)Manβ1-4GlcNAcβ1-4GlcNAcb-Sp20	56	22
406	Galβ1-4GlcNAcβ1-6(Neu5Acα2-6Galβ1-3GlcNAcβ1-3)Galβ1-4Glc-Sp21	56	24
436	Galβ1-6Galb-Sp10	56	16
461	Neu5Acα2-6Galβ1-4GlcNAcβ1-6(Neu5Acα2-6Galβ1-4GlcNAcβ1-2)Manα1-6(GlcNAcβ1-4)(Neu5Acα2-6Galβ1-4GlcNAcβ1-2Manα1-3)Manβ1-4GlcNAcβ1-4GlcNAcb-Sp21	56	14
4	GalNAca-Sp8	56	19
484	Galα1-3Galβ1-3GlcNAcβ1-6GalNAca-Sp14	56	3
387	Galβ1-3GlcNAcβ1-3GalNAca-Sp14	56	19
425	Galβ1-4GlcNAcβ1-6(Fucα1-2Galβ1-3GlcNAcβ1-3)Galβ1-4Glc-Sp21	56	31
16	GlcNAcb-Sp0	56	5
42	(6S)Galβ1-4Glcb-Sp0	56	10
74	Fucα1-2Galβ1-4GlcNAcβ1-3Galβ1-4GlcNAcβ1-3Galβ1-4GlcNAcb-Sp0	56	16
317	Galβ1-4GlcNAcβ1-2Manα1-6(Neu5Acα2-6Galβ1-4GlcNAcβ1-2Manα1-3)Manβ1-4GlcNAcβ1-4GlcNAcb-Sp12	56	8
421	Galα1-3Galβ1-3GlcNAcβ1-3GalNAc-Sp14	56	13
538	GlcNAcβ1-3Galβ1-4GlcNAcβ1-3Galβ1-4GlcNAcβ1-2Manα1-6(GlcNAcβ1-3Galβ1-4GlcNAcβ1-3Galβ1-4GlcNAcβ1-2Manα1-3)Manβ1-4GlcNAcβ1-4GlcNAcb-Sp25	56	33
583	GlcNAcβ1-3Galβ1-4GlcNAcβ1-3GalNAca-Sp14	56	6
22	6S(3S)Galβ1-4(6S)GlcNAcb-Sp0	55	5
277	Neu5Acb2-6Galβ1-4GlcNAcb-Sp8	55	6
516	Galα1-3Galβ1-4GlcNAcβ1-2Mana-Sp0	55	8
17	GlcNAcb-Sp8	55	18
37	(3S)Galβ1-4GlcNAcb-Sp8	55	32
164	Galβ1-4GlcNAcβ1-3Galβ1-4Glcb-Sp0	55	10
347	Galβ1-4GlcNAcβ1-2Manα1-6Manβ1-4GlcNAcβ1-4GlcNAc-Sp12	55	12
173	GlcNAcα1-3Galβ1-4GlcNAcb-Sp8	55	11
30	(3S)Galβ1-3GlcNAcb-Sp0	55	23
129	Galβ1-3(Fucα1-4)GlcNAc-Sp8	55	18
315	Neu5Acα2-3Galβ1-4GlcNAcβ1-6(Neu5Acα2-3Galβ1-3)GalNAca-Sp14	55	12
134	GlcNAcβ1-6(Galβ1-3)GalNAca-Sp14	54	14
246	Neu5Acα2-3Galβ1-3GlcNAcβ1-3Galβ1-4GlcNAcb-Sp0	54	3
364	Neu5Acα2-6GlcNAcβ1-4GlcNAcβ1-4GlcNAc-Sp21	54	14
376	Galβ1-3GalNAcα1-3(Fucα1-2)Galβ1-4Glc-Sp0	54	17
460	Neu5Acα2-6Galβ1-4GlcNAcβ1-4Manα1-6(GlcNAcβ1-4)(Neu5Acα2-6Galβ1-4GlcNAcβ1-4(Neu5Acα2-6Galβ1-4GlcNAcβ1-2)Manα1-3)Manβ1-4GlcNAcβ1-4GlcNAcb-Sp21	54	14
515	Neu5Acα2-6Galβ1-4GlcNAcβ1-2Man-Sp0	54	18
520	Galα1-3(Fucα1-2)Galβ1-3GlcNAcβ1-6GalNAc-Sp14	54	19
527	Galβ1-4GlcNAcβ1-2 Manα1-6(Galβ1-4GlcNAcβ1-4)(Galβ1-4GlcNAcβ1-2Manα1-3)Manβ1-4GlcNAcβ1-4(Fucα1-6)GlcNAc-Sp21	54	5
117	Galα1-3Galβ1-4Glc-Sp10	54	8
276	Neu5Acb2-6GalNAca-Sp8	54	12
344	Neu5Acα2-6Galβ1-4GlcNAcβ1-2Manα1-6Manβ1-4GlcNAcβ1-4GlcNAc-Sp12	54	11
428	GlcNAcβ1-2Manα1-6(GlcNAcβ1-4)(GlcNAcβ1-4(GlcNAcβ1-2)Manα1-3)Manβ1-4GlcNAcβ1-4GlcNAc-Sp21	54	17
87	GalNAcα1-3(Fucα1-2)Galβ1-4Glcb-Sp0	54	26
190	GlcNAcβ1-4GlcNAcβ1-4GlcNAcβ1-4GlcNAcβ1-4GlcNAcβ1-Sp8	54	6
302	Galβ1-4GlcNAcα1-6Galβ1-4GlcNAcb-Sp0	54	8
407	Galβ1-3GalNAcβ1-4(Neu5Acα2-8Neu5Acα2-3)Galβ1-4Glcb-Sp0	53	5
409	Galα1-3(Fucα1-2)Galβ1-4GlcNAcβ1-3GalNAca-Sp14	53	12
493	Galα1-3(Fucα1-2)Galβ1-4GlcNAcβ1-6GalNAca-Sp14	53	10
44	(6S)Galβ1-4GlcNAcb-Sp8	53	19
160	Galβ1-4GlcNAcβ1-3GalNAc-Sp14	53	13
411	GalNAcα1-3GalNAcβ1-3Galα1-4Galβ1-4Glcb-Sp0	53	5
68	Fucα1-2Galβ1-3GlcNAcb-Sp8	53	7
163	Galβ1-4GlcNAcβ1-3Galβ1-4GlcNAcb-Sp0	53	16
279	Neu5Gcα2-3Galβ1-3GlcNAcb-Sp0	53	16

43	(6S)Galβ1-4Glcb-Sp8	53	12
113	Galα1-3Galβ1-4(Fucα1-3)GlcNAcb-Sp8	53	8
557	Galβ1-3GlcNAcβ1-6(Galβ1-3)GalNAc-Sp14	53	5
86	GalNAcα1-3(Fucα1-2)Galβ1-4GlcNAcb-Sp8	52	12
224	Neu5Acα2-3Galβ1-3GalNAca-Sp14	52	16
281	Neu5Gcα2-3Galβ1-4GlcNAcb-Sp0	52	7
92	GalNAcα1-3Galb-Sp8	52	9
231	GalNAcβ1-4(Neu5Acα2-3)Galβ1-4GlcNAcb-Sp8	52	5
361	Galβ1-4GlcNAcβ1-2Manα1-6(Manα1-3)Manβ1-4GlcNAcβ1-4GlcNAcb-Sp12	52	6
433	Galβ1-4GlcNAcβ1-6(Galβ1-4GlcNAcβ1-2)Manα1-6(GlcNAcβ1-4)(Galβ1-4GlcNAcβ1-2Manα1-3)Manβ1-4GlcNAcβ1-4GlcNAc-Sp21	52	9
29	(3S)Galβ1-3GalNAca-Sp8	52	13
594	GlcNAcβ1-6(Neu5Acα2-3Galβ1-3)GalNAca-Sp14	52	18
19	Galβ1-4GlcNAcβ1-6(Galβ1-4GlcNAcβ1-3)GalNAca-Sp8	51	15
410	GalNAcα1-3(Fucα1-2)Galβ1-4GlcNAcβ1-3GalNAca-Sp14	51	3
592	Neu5Acα2-6Galβ1-4GlcNAcβ1-6(Galβ1-3)GalNAca-Sp14	51	10
114	Galα1-3Galβ1-3GlcNAcb-Sp0	51	12
201	GlcAa-Sp8	51	9
341	GlcNAcα1-4Galβ1-3GalNAc-Sp14	51	7
363	Neu5Acα2-6GlcNAcβ1-4GlcNAc-Sp21	51	21
526	Galβ1-4GlcNAcβ1-2 Manα1-6(GlcNAcβ1-4)(Galβ1-4GlcNAcβ1-2Manα1-3)Manβ1-4GlcNAcβ1-4(Fucα1-6)GlcNAc-Sp21	51	9
569	Galβ1-4GlcNAcβ1-3Galβ1-4GlcNAcβ1-3Galβ1-4GlcNAcβ1-3Galβ1-4GlcNAcβ1-2Manα1-6(Galβ1-4GlcNAcβ1-3Galβ1-4GlcNAcβ1-3Galβ1-4GlcNAcβ1-3Galβ1-4GlcNAcβ1-2Manα1-3)Manβ1-4GlcNAcβ1-4(Fucα1-6)GlcNAcb-Sp24	51	13
184	GlcNAcβ1-3Galβ1-4GlcNAcβ1-3Galβ1-4GlcNAcb-Sp0	51	10
221	Fucα1-2Galβ1-4(6S)GlcNAcb-Sp8	51	21
9	Neu5Aca-Sp8	51	17
62	Fucα1-2Galβ1-3GalNAca-Sp14	51	13
508	(3S)GalNAcβ1-4(3S)GlcNAc-Sp8	51	7
511	(4S)GalNAcb-Sp10	51	10
51	Manα1-6(Manα1-3)Manβ1-4GlcNAcβ1-4GlcNAcb-Sp13	50	14
183	GlcNAcβ1-3Galβ1-4GlcNAcb-Sp8	50	10
404	GalNAcβ1-3Galα1-6Galβ1-4Glcb-Sp8	50	11
501	GalNAcα1-3(Fucα1-2)Galβ1-3GlcNAcβ1-6GalNAca-Sp14	50	9
59	Fucα1-2Galβ1-3GalNAcβ1-3Galα1-4Galβ1-4Glcb-Sp9	50	2
137	Neu5Acb2-6(Galβ1-3)GalNAca-Sp8	50	9
587	Neu5Acα2-6Galβ1-4GlcNAcβ1-3Galβ1-4GlcNAcβ1-3GalNAca-Sp14	50	4
472	Neu5Acα2-6Galβ1-4GlcNAcβ1-6(Galβ1-3GlcNAcβ1-3)Galβ1-4Glcb-Sp21	50	10
46	Neu5Acα2-3(6S)Galβ1-4GlcNAcb-Sp8	50	12
382	Galβ1-3GlcNAcβ1-3Galβ1-4(Fucα1-3)GlcNAcβ1-6(Galβ1-3GlcNAcβ1-3)Galβ1-4Glc-Sp21	50	8
573	GlcNAcβ1-3Galβ1-4GlcNAcβ1-3Galβ1-4GlcNAcβ1-6(GlcNAcβ1-3Galβ1-4GlcNAcβ1-3Galβ1-4GlcNAβ1-2)Manα1-6(GlcNAcβ1-3Galβ1-4GlcNAcβ1-3Galβ1-4GlcNAcβ1-2Manα1-3)Manβ1-4GlcNAcβ1-4(Fucα1-6)GlcNAcb-Sp24	49	9
591	Neu5Acα2-6Galβ1-4GlcNAcβ1-3Galβ1-4GlcNAcβ1-6(Galβ1-3)GalNAca-Sp14	49	12
12	Galb-Sp8	49	8
503	Galβ1-4GlcNAcβ1-6(Galβ1-4GlcNAcβ1-2)Manα1-6(GlcNAcβ1-4)Galβ1-4GlcNAcβ1-4(Gal β1-4GlcNAcβ1-2)Manα1-3)Manβ1-4GlcNAcβ1-4(Fucα1-6)GlcNAc-Sp21	49	6
66	Fucα1-2Galβ1-3GlcNAcβ1-3Galβ1-4Glcb-Sp10	49	6
458	Neu5Acα2-3Galβ1-4GlcNAcβ1-6(Neu5Acα2-3Galβ1-4GlcNAcβ1-2)Manα1-6(GlcNAcβ1-4)(Neu5Acα2-3Galβ1-4GlcNAcβ1-4(Neu5Acα2-3Galβ1-4GlcNAcβ1-2)Manα1-3)Manβ1-4GlcNAcβ1-4GlcNAcb-Sp21	49	10
61	Fucα1-2Galβ1-3GalNAca-Sp8	49	10
372	Neu5Acα2-3Galβ1-4GlcNAcβ1-3GalNAc-Sp14	49	6
494	Fucα1-2Galβ1-4GlcNAcβ1-2Mana-Sp0	49	19
242	Neu5Acα2-6(Neu5Acα2-3Galβ1-3)GalNAca-Sp8	48	12
499	(3S)GalNAcβ1-4(Fucα1-3)GlcNAcb-Sp8	48	4
223	Neu5Acα2-3Galβ1-3GalNAca-Sp8	48	19

457	Neu5Acα2-3Galβ1-4GlcNAcβ1-6(Neu5Acα2-3Galβ1-4GlcNAcβ1-2)Manα1-6(GlcNAcβ1-4)(Neu5Acα2-3Galβ1-4GlcNAcβ1-2Manα1-3)Manβ1-4GlcNAcβ1-4GlcNAcb-Sp21	48	6
491	Galβ1-4(Fucα1-3)GlcNAcβ1-2Mana-Sp0	48	10
25	(3S)Galβ1-4Glcb-Sp8	48	15
138	Neu5Acα2-6(Galβ1-3)GlcNAcβ1-4Galβ1-4Glcb-Sp10	48	19
559	(3S)GlcAβ1-3Galβ1-4GlcNAcβ1-3Galβ1-4Glc-Sp0	48	11
10	Neu5Aca-Sp11	47	8
146	Galβ1-3Galb-Sp8	47	11
291	Galβ1-4GlcNAcβ1-3Galβ1-3GlcNAcb-Sp0	47	13
578	Galβ1-4GlcNAcβ1-3Galβ1-4GlcNAcβ1-3Galβ1-4GlcNAcβ1-3Galβ1-4GlcNAcβ1-3Galβ1-4GlcNAcβ1-6(Galβ1-4GlcNAcβ1-3Galβ1-4GlcNAcβ1-3Galβ1-4GlcNAcβ1-3Galβ1-4GlcNAcβ1-3Galβ1-4GlcNAβ1-2)Manα1-6(Galβ1-4GlcNAcβ1-3Galβ1-4GlcNAcβ1-3Galβ1-4GlcNAcβ1-3Galβ1-4GlcNAcβ1-3Galβ1-4GlcNAcβ1-2Manα1-3)Manβ1-4GlcNAcβ1-4(Fucα1-6)GlcNAcb-Sp24	47	9
588	GlcNAcβ1-3Galβ1-4GlcNAcβ1-3Galβ1-4GlcNAcβ1-3GalNAca-Sp14	47	8
118	Galα1-3Galb-Sp8	47	9
545	Neu5Gcα2-8Neu5Gcα2-3Galβ1-4GlcNAcβ1-3Galβ1-4GlcNAc-Sp0	47	4
560	(3S)GlcAβ1-3Galβ1-4GlcNAcβ1-2Mana-Sp0	47	6
275	Galβ1-3(Fucα1-4)GlcNAcβ1-3Galβ1-3(Fucα1-4)GlcNAcb-Sp0	47	15
26	(3S)Galβ1-4(6S)Glcb-Sp0	46	12
483	Galβ1-3GlcNAcβ1-6GalNAca-Sp14	46	1
547	Neu5Acα2-8Neu5Acα2-3Galβ1-4GlcNAc-Sp0	46	5
552	GalNAcβ1-3GlcNAcb-Sp0	46	7
48	Neu5,9Ac2a-Sp8	46	16
112	Galα1-3GalNAcb-Sp8	46	9
147	Galβ1-3GlcNAcβ1-3Galβ1-4GlcNAcb-Sp0	46	18
432	Galβ1-4GlcNAcβ1-2Manα1-6(GlcNAcβ1-4)(Galβ1-4GlcNAcβ1-4(Galβ1-4GlcNAcβ1-2)Manα1-3)Manβ1-4GlcNAcβ1-4GlcNAc-Sp21	46	13
513	(6P)Galβ1-4GlcNAcb-SP0	46	10
198	Glcβ1-4Glcb-Sp8	46	6
440	(6S)Galβ1-3(6S)GlcNAc-Sp0	46	10
27	(3S)Galβ1-4(6S)Glcb-Sp8	45	9
264	Neu5Acα2-6GalNAcβ1-4GlcNAcb-Sp0	45	14
319	Neu5Acα2-8Neu5Acα2-8Neu5Acb-Sp8	45	11
383	Galβ1-4GlcNAcβ1-6(Galβ1-4GlcNAcβ1-2)Manα1-6(Galβ1-4GlcNAcβ1-4(Galβ1-4GlcNAcβ1-2)Manα1-3)Manβ1-4GlcNAcβ1-4GlcNAcb-Sp21	45	17
438	GalNAcβ1-6GalNAcb-Sp8	45	16
152	Galβ1-4(Fucα1-3)GlcNAcb-Sp8	45	6
479	Manα1-6(Manα1-3)Manβ1-4GlcNAcβ1-4(Fucα1-6)GlcNAcb-Sp19	45	9
274	Neu5Acα2-8Neu5Acα2-3Galβ1-4Glcb-Sp0	45	12
530	GlcNAcβ1-3Galβ1-4GlcNAcβ1-6(GlcNAcβ1-3)Galβ1-4GlcNAc-Sp0	45	14
39	(6S)(4S)Galβ1-4GlcNAcb-Sp0	44	19
316	Neu5Acα2-6Galβ1-4GlcNAcβ1-2Manα1-6(Neu5Acα2-3Galβ1-4GlcNAcβ1-2Manα1-3)Manβ1-4GlcNAcβ1-4GlcNAcb-Sp12	44	14
389	GalNAcα1-3(Fucα1-2)Galβ1-3GalNAcα1-3(Fucα1-2)Galβ1-4GlcNAcb-Sp0	44	18
431	Galβ1-4GlcNAcβ1-2Manα1-6(GlcNAcβ1-4)(Galβ1-4GlcNAcβ1-2Manα1-3)Manβ1-4GlcNAcβ1-4GlcNAc-Sp21	44	23
514	GalNAcα1-3(Fucα1-2)Galβ1-4GlcNAcβ1-6GalNAc-Sp14	44	13
18	GlcN(Gc)b-Sp8	44	25
181	GlcNAcβ1-3Galb-Sp8	44	11
45	(6S)Galβ1-4(6S)Glcb-Sp8	44	13
456	Neu5Acα2-3Galβ1-4GlcNAcβ1-4Manα1-6(GlcNAcβ1-4)(Neu5Acα2-3Galβ1-4GlcNAcβ1-4(Neu5Acα2-3Galβ1-4GlcNAcβ1-2)Manα1-3)Manβ1-4GlcNAcβ1-4GlcNAcb-Sp21	43	11
455	Neu5Acα2-3Galβ1-4GlcNAcβ1-2Manα1-6(GlcNAcβ1-4)(Neu5Acα2-3Galβ1-4GlcNAcβ1-2Manα1-3)Manβ1-4GlcNAcβ1-4GlcNAcb-Sp21	43	23
577	GlcNAcβ1-3Galβ1-4GlcNAcβ1-3Galβ1-4GlcNAcβ1-3Galβ1-4GlcNAcβ1-3Galβ1-4GlcNAcβ1-6(GlcNAcβ1-3Galβ1-4GlcNAcβ1-3Galβ1-4GlcNAcβ1-3Galβ1-4GlcNAcβ1-3Galβ1-4GlcNAβ1-2)Manα1-6(GlcNAcβ1-3Galβ1-4GlcNAcβ1-3Galβ1-4GlcNAcβ1-3Galβ1-4GlcNAcβ1-3Galβ1-4GlcNAcβ1-2Manα1-3)Manβ1-4GlcNAcβ1-4(Fucα1-6)GlcNAcb-Sp24	43	10
21	GlcNAcβ1-6(GlcNAcβ1-4)(GlcNAcβ1-3)GlcNAc-Sp8	42	14
186	GlcNAcβ1-4-MDPLys	42	8
158	Galβ1-4GalNAcβ1-3(Fucα1-2)Galβ1-4GlcNAcb-Sp8	42	10
392	Neu5Acα2-3Galβ1-3GlcNAcβ1-2Manα1-6(Neu5Acα2-3Galβ1-3GlcNAcβ1-2Manα1-3)Manβ1-4GlcNAcβ1-4GlcNAc-Sp19	42	14

140	Galβ1-3GalNAca-Sp14	42	18
574	Galβ1-4GlcNAcβ1-3Galβ1-4GlcNAcβ1-3Galβ1-4GlcNAcβ1-6(Galβ1-4GlcNAcβ1-3Galβ1-4GlcNAcβ1-3Galβ1-4GlcNAβ1-2)Manα1-6(Galβ1-4GlcNAcβ1-3Galβ1-4GlcNAcβ1-3Galβ1-4GlcNAcβ1-2Manα1-3)Manβ1-4GlcNAcβ1-4(Fucα1-6)GlcNAcb-Sp24	41	8
459	Neu5Acα2-6Galβ1-4GlcNAcβ1-2Manα1-6(GlcNAcβ1-4)(Neu5Acα2-6Galβ1-4GlcNAcβ1-2Manα1-3)Manβ1-4GlcNAcβ1-4GlcNAcb-Sp21	41	9
492	Fucα1-2(6S)Galβ1-3GlcNAcb-Sp0	41	13
570	GlcNAcβ1-3Galβ1-4GlcNAcβ1-3Galβ1-4GlcNAcβ1-3Galβ1-4GlcNAcβ1-3Galβ1-4GlcNAcβ1-2Manα1-6(GlcNAcβ1-3Galβ1-4GlcNAcβ1-3Galβ1-4GlcNAcβ1-3Galβ1-4GlcNAcβ1-3Galβ1-4GlcNAcβ1-2Manα1-3)Manβ1-4GlcNAcβ1-4(Fucα1-6)GlcNAcb-Sp19	41	9
49	Neu5,9Ac2α2-6Galβ1-4GlcNAcb-Sp8	41	7
135	Neu5Acα2-6(Galβ1-3)GalNAca-Sp8	41	12
595	Neu5Acα2-6Galβ1-4GlcNAcβ1-3Galβ1-4GlcNAcβ1-6(Neu5Acα2-6Galβ1-4GlcNAcβ1-3Galβ1-4GlcNAcβ1-3)GalNAca-Sp14	41	8
2	Glca-Sp8	41	22
171	Galβ1-4Glcb-Sp0	41	5
589	Galβ1-4GlcNAcβ1-3Galβ1-3GalNAca-Sp14	40	12
210	Manα1-6(Manα1-2Manα1-3)Manα1-6(Manα1-2Manα1-3)Manβ1-4GlcNAcβ1-4GlcNAcb-Sp12	40	8
540	Galβ1-4GlcNAcβ1-3Galβ1-4GlcNAcβ1-3Galβ1-4GlcNAcβ1-2Manα1-6(Galβ1-4GlcNAcβ1-3Galβ1-4GlcNAcβ1-3Galβ1-4GlcNAcβ1-2Manα1-3)Manβ1-4GlcNAcβ1-4GlcNAcb-Sp24	40	9
14	Manb-Sp8	39	4
169	Galβ1-4GlcNAcb-Sp8	39	6
232	GalNAcβ1-4(Neu5Acα2-3)Galβ1-4Glcb-Sp0	39	4
85	GalNAcα1-3(Fucα1-2)Galβ1-4GlcNAcb-Sp0	39	22
139	Galβ1-3GalNAca-Sp8	39	9
241	Neu5Acα2-3Galβ1-3(6S)GalNAca-Sp8	38	13
58	Fucα1-2Galβ1-3GalNAcβ1-3Gala-Sp9	38	11
106	Galα1-3(Fucα1-2)Galβ1-4Glcb-Sp0	37	8
252	Neu5Acα2-3Galβ1-4(Fucα1-3)GlcNAcb-Sp0	37	12
437	Neu5Acα2-3Galβ1-4GlcNAcβ1-3Galb-Sp8	37	13
338	GlcNAcα1-4Galβ1-4GlcNAcβ1-3Galβ1-4Glcb-Sp0	37	11
38	(3S)Galb-Sp8	37	12
505	Galβ1-3(6S)GlcNAcb-Sp8	37	9
435	Galβ1-4Galb-Sp10	36	8
265	Neu5Acα2-6Galβ1-4(6S)GlcNAcb-Sp8	36	24
15	GalNAcb-Sp8	35	13
497	Neu5Acα2-6GalNAcβ1-4(6S)GlcNAcb-Sp8	35	20
504	Galβ1-3GlcNAcα1-3Galβ1-4GlcNAcb-Sp8	35	11
490	Galα1-3Galβ1-4GlcNAcβ1-6GalNAca-Sp14	34	11
125	Galβ1-2Galb-Sp8	34	12
481	Neu5Acα2-3Galβ1-3GlcNAcβ1-2Manα1-6(GlcNAcβ1-4)(Neu5Acα2-3Galβ1-3GlcNAcβ1-2Manα1-3)Manβ1-4GlcNAcβ1-4GlcNAc-Sp21	34	4
544	Neu5Gcα2-8Neu5Acα2-3Galβ1-4GlcNAc-Sp0	33	16
1	Gala-Sp8	33	22
507	(6S)GalNAcβ1-4GlcNAc-Sp8	33	3
575	GlcNAcβ1-3Galβ1-4GlcNAcβ1-3Galβ1-4GlcNAcβ1-3Galβ1-4GlcNAcβ1-6(GlcNAcβ1-3Galβ1-4GlcNAcβ1-3Galβ1-4GlcNAcβ1-3Galβ1-4GlcNAβ1-2)Manα1-6(GlcNAcβ1-3Galβ1-4GlcNAcβ1-3Galβ1-4GlcNAcβ1-3Galβ1-4GlcNAcβ1-2Manα1-3)Manβ1-4GlcNAcβ1-4(Fucα1-6)GlcNAcb-Sp24	33	18
506	(6S)(4S)GalNAcβ1-4GlcNAc-Sp8	32	8
5	GalNAca-Sp15	32	7
41	(6P)Mana-Sp8	31	14
576	Galβ1-4GlcNAcβ1-3Galβ1-4GlcNAcβ1-3Galβ1-4GlcNAcβ1-3Galβ1-4GlcNAcβ1-6(Galβ1-4GlcNAcβ1-3Galβ1-4GlcNAcβ1-3Galβ1-4GlcNAcβ1-3Galβ1-4GlcNAβ1-2)Manα1-6(Galβ1-4GlcNAcβ1-3Galβ1-4GlcNAcβ1-3Galβ1-4GlcNAcβ1-3Galβ1-4GlcNAcβ1-2Manα1-3)Manβ1-4GlcNAcβ1-4(Fucα1-6)GlcNAcb-Sp24	30	8
564	GlcNAcβ1-3Galβ1-4GlcNAcβ1-2Manα1-6(GlcNAcβ1-3Galβ1-4GlcNAcβ1-2Manα1-3)Manβ1-4GlcNAcβ1-4(Fucα1-6)GlcNAcb-Sp24	30	5
542	Neu5Gcα2-8Neu5Gcα2-3Galβ1-4GlcNAc-Sp0	29	5
543	Neu5Acα2-8Neu5Gcα2-3Galβ1-4GlcNAc-Sp0	28	8
567	Galβ1-4GlcNAcβ1-3Galβ1-4GlcNAcβ1-3Galβ1-4GlcNAcβ1-2Manα1-6(Galβ1-4GlcNAcβ1-3Galβ1-4GlcNAcβ1-3Galβ1-4GlcNAcβ1-2Manα1-3)Manβ1-4GlcNAcβ1-4(Fucα1-6)GlcNAcb-Sp24	27	12

558	Galβ1-4GlcNAcβ1-3Galβ1-4GlcNAcβ1-3Galβ1-4GlcNAcβ1-3Galβ1-4GlcNAcβ1-3Galβ1-4GlcNAcβ1-3Galβ1-4GlcNAcβ1-2Manα1-6(Galβ1-4GlcNAcβ1-3Galβ1-4GlcNAcβ1-3Galβ1-4GlcNAcβ1-3Galβ1-4GlcNAcβ1-3Galβ1-4GlcNAcβ1-3Galβ1-4GlcNAcβ1-2Manα1-3)Manβ1-4GlcNAcβ1-4GlcNAcb-Sp25	27	7
6	Fuca-Sp8	26	15
546	Neu5Gcα2-8Neu5Gcα2-6Galβ1-4GlcNAc-Sp0	25	7
8	Rhaa-Sp8	24	14
348	Manα1-6(Galβ1-4GlcNAcβ1-2Manα1-3)Manβ1-4GlcNAcβ1-4GlcNAcb-Sp12	18	30
3	Mana-Sp8	15	22
502	GlcNAcβ1-6(GlcNAcβ1-2)Manα1-6(GlcNAcβ1-4)(GlcNAcβ1-4(GlcNAcβ1-2)Manα1-3)Manβ1-4GlcNAcβ1-4(Fucα1-6)GlcNAc-Sp21	12	5

Figure S1. Sequences of C-type lectin-like domains encoded in CD23 genes in mammals. Sequences are shown for all mammalian genes that display the conserved framework residues highlighted in *yellow*. Potential ligands for the conserved, principal Ca^{2+} site are highlighted in *green* and potential ligands for the accessory Ca^{2+} site are highlighted in *magenta*. Residues that do not conform to these patterns are highlighted in *gray*.

Homo sapiens (human)	YYFGKGT-KQWVHARYACDDMEGQLVSIHSPEEQDFLTKHASHTGSWIGLRNLDLKGEFIWVDGSHVDYSNWAPGEPTSRSQGEDCVMMRGS-GRWNDAFCDRKLGAWVCDRLATCTPPASEGSAESMGPDSRPDPDGRLPTPSAPLHS
Pan troglodytes (chimpanzee)	YYFGKGT-KQWVHARYACDDMEGQLVSIHSPEEQDFLTKHASHTGSWIGLRNLDLKGEFIWVDGSHVDYSNWAPGEPTSRSQGEDCVMMRGS-GRWNDAFCDRKLGAWVCDRLATCTPPASEGSAESMGPDSRPGPDGRLPAPSAPLHS
Pan paniscus (pygmy chimpanzee)	YYFGKGT-KQWVHARYACDDMEGQLVSIHSPEEQDFLTKHASHTGSWIGLRNLDLKGEFIWVDGSHVDYSNWAPGEPTSRSQGEDCVMMRGS-GRWNDAFCDRKLGAWVCDRLATCTPPASEGSAESMGPDSRPGPDGRLPAPSAPLHS
Nomascus leucogenys (northern white-cheeked gibbon)	YYFGKGT-KQWVHARYACEDMEGQLVSIHSLEEQDFLTKHASHTGSWIGLRNLDLKGEFVWVDGSHVDYSNWAPGEPTSRSQGEDCVMMRGS-GHWNDAFCDRELGAWVCDRLATCTPPASEGSAESVGPDSRRGPDGRLPAPSAPLHS
Cebus capucinus imitator (white-headed capuchin)	YYFGKGT-KQWVHARYACEDMEGQLVSIHSHEEQDFLIKHASHTGSWIGLRNLDLKGEFIWMDGTPVDYSNWAPEEPTNRNQGEDCVMMRGS-GDWNDIYCDRKLGAWVCDRLATCVPLDREGSAEFLEPDSELGPDSEPGQDGRLPIPSALLHP
Callithrix jacchus (white-tufted-ear marmoset)	YYFGKGT-KQWVHARYACEDMEGQLVSIHSHEEQDFLIKHASRTGSWIGLRNLDLKGEFIWMDGTPVDYSNWAPEEPTNRNQGEDCVMMRGS-GDWNDIYCDRKLGTWVCDRLATCTLPAREGSAESPGPDSEPDQNGRLPTPLPLSTLEHGPSQPRVKP
Saimiri boliviensis (Bolivian squirrel monkey)	YYFGKGT-KQWVHARYACEDVEGQLVSIHSQEEQDFLIKHASRTGSWIGLRNLDLKGEFIWMDGTPVDYSNWAPEEPTNRNQGEDCVMMRGS-GDWNDIYCDRKLGAWVCDRLATCVPPAREGSAEPPEPGPDSEPGQDDRLPIPSALLHP
Gorilla gorilla (western gorilla)	YYFGKGT-KQWVHARYACDDMEGQLVSIHSPEEQDFLTKHASHTGSWIGLRNLDLKGEFIWVDGSHVDYSNWAPGEPTSRSQGKDCVMMRGS-GRWNDAFCHRKLGAWVCDRLATCTPPASEGSAESMGPDSRPGPDGRLPAPSAPLHS
Pongo abelii (Sumatran orangutan)	YYFGKGT-KQWVHARYACDDMEGQLVSIHSPEEQDFLTKHASHIGSWIGLRNLDLKGEFIWVDGSHVDYSNWAPGEPTSRSQGEDCVMMRGS-GHWHDAFCDRKLGTWVCDRLATCTPPASEGSAESVGPDSRPGPDGRLPAPSAPLRS"
Rhinopithecus bieti (black snub-nosed monkey)	YYFGKGT-KQWLHARYACEDMEGQLVSIHSPEEQDFLTKHASHTGSWIGLRNLNLKGEFIWVDGSPVDYSNWAPGEPTSQSQGKDCVMMRGS-SHWNNAFCDRKLGAWVCDRLATCAPPASEGSTESLGPESRSSPDGRLPAPSAPLHS
Rhinopithecus roxellana (golden snub-nosed monkey)	YYFGKGT-KQWLHARYACEDMEGQLVSIHSPEEQDFLTKHASHTGSWIGLRNLNLKGEFIWVDGSPMDYSNWAPGEPTSQSQGKDCVMMRGS-SHWNNAFCDRKLGAWVCDRLATCAPPASEGSTESLGPESRSSPDGRLPAPSAPLHS
Piliocolobus tephrosceles (Ugandan red Colobus)	YYFGKGT-KQWLHARYACEDMEGQLVSIHSPEEQDFLTKHASRTGSWIGLRNLNLKGEFIWVDGSPVDYSNWAPGEPTSQSQGKDCVMMRGS-SHWNNAFCDRKLGAWVCDRLATCVPPASEGSTESLGPESRSGPDGRLPAPSAPLHS
Colobus angolensis palliatus (Angolan Colobus)	YYFGKGT-KQWLHARYACEDVEGQLVSIHSPEEQDFLTKHASRTGSWIGLRNLNLKGEFIWVDGSPVDYSNWAPGEPTSQSQGKDCVMMRGS-SHWNNAFCDRKLGAWVCDRLATCAPPASEGSTESLGPESRSGPDGRLPAPSAPLHS
Chlorocebus sabaeus (green monkey)	YYFGKGT-KQWLHARYACEDMEGQLVSIHSPEEQDFLTKHASHTISWIGLRNLNLKGEFIWVDGSPVDYSNWAPGEPTNQSHGKDCVMMRGS-GHWNNAFCDRKLGSWVCDRMATCAPPANEGSAESLGPESRPGP
Macaca mulatta (Rhesus monkey)	YYFGKGT-KQWLHARYACEDMEGQLVSIHSPEEQDFLTKHASHTISWIGLRNLNLKGEFIWVDGSPVDYSNWAPGEPASQSHGKDCVMMRGS-SHWNNAFCDRKLGSWVCDRLATCAPPANEGSAESLGPESRPGPDGRLSAPSAPLHS
Theropithecus gelada (gelada)	YYFGKGT-KQWLHARYACEDMEGQLVSIHSPEEQDFLTKYASHTISWIGLRNLNLKGEFIWVDGSPVDYSNWAPGEPASQSHGKDCVMMRGS-SHWNNAFCDRKLGSWVCERLATCAPPANEGSAESLGPESRPGP
Macaca nemestrina (pig-tailed macaque)	YYFGKGT-KQWLHARYACEDMEGQLVSIHSPEEQDFLTKHASHTISWIGLRNLNLKGEFIWVDGSPVDYSNWAPGEPASQSHGKDCVMMRGS-SHWNNAFCDRKLGSWVCDRLATCAPPANEGSAESLGPESRPGPDGRLSAPSAPLHS
Mandrillus leucophaeus (drill)	YYFGKGT-KQWLHARYACEDMEGQLVSIHSPEEQDFLTKHASHTISWIGLRNLNLKGEFIWVDGSPVDYSNWAPGEPASQSHGKDCVMMRGS-SHWNNAFCDRKLGSWVCDRLATCAPPASEGSAESLGPESRPGP
Cercocebus atys (sooty mangabey)	YYFGKGT-KQWLHARYACEDMEGQLVSIHSPEEQDFLTKHASHTISWIGLRNLNLKGEFIWVDGSPVDYSNWAPGEPASQSHGKDCVMMRGS-SHWNNAFCDRKLGSWVCDRLATCAPPASEGSAESLGPESRPGP
Carlito syrichta (Philippine tarsier)	YYFGEGA-KQWIQARYACSDVGGRLVSIHSPEEQDFLTKHASKKGSWIGLRDLDVEGEFIWMDGSPVDYSNWLPGEPNNHGQGEDCVMMQGS-GHWNDILCRSKLDAWVCDRLATCTPSTASASQAPPGPALRQRS
Propithecus coquereli (Coquerel's sifaka)	YYFGEGA-KKWLHAQYACEDLGGRLASIHSAEEQDFLSKRANWKGSWIGLQDLDREGQFTWMDGSPMGYSNWYPGEPNNQGQGEDCVMMRGS-GQWNDAACHSKLDSWVCDRLATCGPPATSTPEGPPGTTPSL
Microcebus murinus (grey mouse lemur)	YYFGEGA-KKWLHAQYACEDVGGRLASIHGAEEQDFLSKRASWKGSWIGLQDLDREGQFTWMDGTPMDYSNWSPGEPNNQAQGEDCVMMRGS-GQWNDAACHSKLDAWVCDRLATCGPPTTSTPEGPPGTTPSL
Otolemur garnettii (small-eared galago / bushbaby)	YYFGEGA-KKWLHAQYACEAMGGRLVSIHSREEQDFLIKRADWKGSWIGLQDLDREGQFTWMDGSPVSYSNWYPGEPNNQDQGENCVMMRGS-GQWNDAACQSRLDAWVCEQLATCGLPATLVPVEPLGTAPSPEP
Mus musculus (house mouse)	YYFGKGS-KQWIQARFACSDLQGRLVSIHSQKEQDFLMQHINKKDSWIGLQDLNMEGEFVWSDGSPVGYSNWNPGEPNNGGQGEDCVMMRGS-GQWNDAFCRSYLDAWVCEQLATCEISAPLASVTPTRPTPKSEP
Mus caroli (Ryukyu mouse)	YYFGKGS-KQWIQARFTCSDLQGRLVSIHSQKEQDFLMQHINKKDSWIGLQDLNMEGEFVWSDGSPVGYSNWNPGEPNNGGQGEDCVMMRGS-GHWNDAFCRSYLDAWVCEQLATCELSAPLASVTPTRPTPKSEP
Rattus norvegicus (rat)	YYFGEGS-KQWIQAKFTCSDLEGRLVSIHSQKEQDFLMQHINKKESWIGLQDLNMEGEFVWPDGSPVGYSNWSPGEPNNGGQGEDCVMMRGS-GQWNDAFCRSYLDAWVCEQLATCDLSAPLASVTPTGPTPKNEP
Microtus ochrogaster (prairie vole)	YYFGNDS-KRWIQARFTCSDLKGRLVSIHSQEEQNFLMKHTNKD-SWIGLRDLNVEGEFIWMDGSPVGYSNWSPGEPNNAGQGEDCVMMRGS-GQWNDAFCHSFLEGWVCEQLATCEDSAPLASERPTGPTTPKTEP
Peromyscus maniculatus bairdii (prairie deer mouse)	YYFGKGS-KQWIQARFTCSDLEGRLVSIHSQEEQDFLMRHINKQDSWIGLRDLNVEGEFVWMDGSPVGYSNWNPGEPNNAGQGEDCVMMRGS-GQWNDAYCRSYLDAWVCEQLATCEEASAPVASLSPTGPTPKNGS
Peromyscus leucopus (white-footed mouse)	YYFGKGP-KQWIQARFTCSDLEGRLVSIHSQEEQDFLMRHINKQDSWIGLRDLNVEGEFVWMDGSPVGYSNWNPGEPNNAGQGEDCVMMRGS-GQWNDAYCRSYLDAWVCEQLATCEEASAPVVSLSPTGPTPKNGS
Crisetulus griseus (Chinese hamster)	YYFGKGP-KQWIQARFACSDLEGRLVSIHSQEEQDFLMKHISKD-SWIGLRDLNVEGQFIWMDGSPVGYSNWSPGEPNNAEQGEDCVLMRES-GQWNDYSCRSFLYAWVCEQLATCEASAPLASVSPTGPTPSNEP
Misocricetus auratus (golden hamster)	YYFGKGS-KQWIQARFACSDLEGRLVSIHSQEEQDFLMKHINKD-AWIGLRDLNVEGQFIWMDGSPVGYSNWSPGEPNNAEQGEDCVMMRGS-GQWNDNSCRSFLDAWVCEQLATCEASAPSASVSPTGPTPSNEP
Heterocephalus glaber (naked mole-rat)	YYFGEGP-KQWVQARFTCNDLEGRLVSIHSQEEQDFLTKHANKEGSWIGLRDLDIEGEFTWMDGSPLDYSNWQSGEPNNA-QGEDCVVMQGS-GQWNDAFCRTWLDAWVCEQLATCGPPTPSDSTDDSPGSGSTHSP
Fukomys damarensis (Damara mole-rat)	YYFGEGA-KQWVQARFTCDDLEGRLVSIHSQEEQDFLTKHANKEGSWIGLRDLDVEGEFIWMDGSPLDYSNWQSGEPNNA-QGEDCVMMQGS-GKWNDAFCRSWLDAWVCEQLATCGSPTPSDATDDSRVSGLGSGSAHSP
Marmota marmota marmota (Alpine marmot)	YYFGQGA-KRWVQARFTCDDLEGRLVSIHSQEEQDFLTKRANKKGSWIGLRDLDLEGEFIWMDGSPLDYSNWNLGEPNNGDQGEDCVMMLGS-GKWNDASCRSELDAWVCEQLATCGPPAPSASGDVLEPVPTEGH
Marmota flaviventris (Yellow-bellied marmot)	YYFGQGA-KRWVQARFTCDDLEGRLVSIHSQEEQDFLTKRASKKGSWIGLRDLDLEGEFIWMDGSPLDYSNWNLGEPNNGDQGEDCVMMLGS-GKWNDASCRSELDAWVCEQLATCGPPAPSASGDVLEPVPTEGH
Ictidomys tridecemlineatus (13-lined ground squirrel)	YYFGQGA-KRWVQARFTCDDLEGRLVSIHSQEEQDFLTKRANKRGSWIGLRDLDLEGEFIWMDGSPLDYSNWNLGEPNNGDQGEDCVMMLGS-GKWNDASCRGELDAWVCEQLATCGPPAPSASVTPPKPSPRVTPAPDNYSC
Urocitellus parryii (Arctic ground squirrel)	YYFGQGA-KRWVQAKFTCDDLEGRLVSIHSQEEQDFLTKHANKRGSWIGLRDLDLEGEFIWMDGSPLGYSNWNLGEPNNGDQGEDCVMMLGS-GKWNDASCRGELDAWVCEQLATCGPPAPSASVTPPKPSPRVTPAPDNYSVTTCLDSHGSRKCSSGSSRNANS
Nannospalax galili (upper Galilee mtn blind mole rat)	YYFGKGS-KQWIQAKFTCSNLKGQLVSIHSQEEQDFLTKHIKED-SWIGLRDLFTEGEFVWMDDSPVGYSNWNPGEPNNGGQGEDCVMMWGS-GQWNDAFCRSYLESWVCEQLATCEPSAPLASMSPLSSAEPAS
Cavia porcellus (domestic guinea pig)	YYFGESP-KRWLQARYACEDLHGRLVSIHSQEEQDFLTKRANKEKAWIGLRDLDIEGEFTWMDGKPLDYTNWQPGEPNNADQGENCVMMLSS-GKWNDAFCHNWLDAWVCEQLATCGSPATSDSLDDSLDTGST
Chinchilla lanigera (long-tailed chinchilla)	YYFGKGP-KQWIQARFSCEDLEGRLVSIHSQEEQDFLTKHANKEDSWIGLRDLDIEGEFIWTDGRPVDYSNWQPGEPNNADQGEHCVMMLSS-GGWNDAFCRSQLDAWVCEQLAACGSAPPSDPTDDSVDAGSASL
Octodon degus (degu)	YYFGEEP-KQWLQARYSCEDLGGRLVSIHSQEQQDFLTKLAKKEGTWIGLRDLDIEGQFLWMDGSSVDYSNWQPGEPNNADPGEDCVMLLRS-GKWNDAFCRDELDAWVCEQLATCGPSFPADPTDDSLDSDSLYSGST
Castor canadensis (American beaver)	YYFGKSA-KQWIQARXACSDLEGRLVSIHSQEEQDFLTKHANKKGSWIGLRDLDVEGKFIWMDGTPVDYSNWHPGEPNNEGQGEDCVMMQGS-GQWNDAFCRSQLDAWVCEQLATCSPSAPSASAEPALTHGPGLPSSTPS
Dipodomys ordii (Ord's kangaroo rat)	YYFGEGT-KRWLQARFACSDLEGR LVSIHSQEEQDFLSKHVSKDAWIGLQDLDVEGEFVWMDGSPVSYSNWLQGEPNNAGSDEDCVMMRGS-GHWNDAFCRSQLDTWVCERLATCGPPALPTSGTPTPRHTP
Bos taurus (cow)	YYFGEGA-KKWIQARYACENLHGRLVSIHSPEEQDFLTKRANWRGSWIGLRDLDIEGEFIWMDNQPLDYSNWQPGEPNDAGQGENCVMMLGS-GKWNDAFCGSELHGWVCDRLATC
Bos indicus (zebu)	YYFGEGA-KKWIQARYACENLHGRLVSIHSPEEQDFLTKRANWRGSWIGLRDLDIEGEFIWMDNQPLDYSNWQPGEPNDAGQGENCVMMLGS-GKWNDAFCGSELHGWVCDRLATC
Bos indicus x *Bos taurus* (hybrid cattle)	YYFGEGA-KKWIQARYACENLHGRLVSIHSPEEQDFLTKRANWRGSWIGLRDLDIEGEFIWMDNQPLDYSNWQPGEPNDAGQGENCVMMLGS-GKWNDAFCGSELHGWVCDRLATC
Bos mutus (wild yak)	YYFGEGA-KKWIQARYACENLHGRLVSIHSPEEQDFLTKRANWRGSWIGLRDLDIEGEFIWMDNQPLDYSNWQPGEPNDAGQGENCVMMLGS-GKWNDAFCGSELHGWVCDRLATC
Ovis aries (sheep)	YYFGEGA-KKWIQARYACEDLDGRLVSIHSPEEQDFLTKRASWRGSWIGLRDLDIEGEFIWMDNQPLDYSNWQPGEPNDGGQGENCVMMLGS-GKWNDAFCGSDLHGWVCDRLATC
Capra hircus (goat)	YYFGEGA-KKWIQARYACEDLDGRLVSIHSPEEQDFLTKRANWRGSWIGLRDLDIEGEFIWMDNQPLDYSNWQPGEPNDGGQGENCVMMLGS-GKWNDAFCGSDLHGWVCDRLATC
Bubalus bubalis (water buffalo)	YYFGEGA-KKWIQARYACENLHGRLVSIHSPEEQDFLTKRANWRGSWIGLRDLDIEGEFIWMDNQPLDYSNWQPGEPNDAGQGENCVMMLGS-GKWNDAFCGSELHGWVCDRLATC
Pantholops hodgsonii (chiru / Tibetan antelope)	YYFGEGA-KKWIQARYACEDLDGRLVSIHSPEEQGFLTKRANWRGSWIGLRDLDIEGEFIWMDNQPLDYSNWQPGEPNDGGQGENCVMMLGS-GKWNDAFCGSHLHGWVCDRLATC
Balaenoptera acutorostrata scammoni (Minke whale)	YYFGEGT-KKWIQARNACSKLHGRLVSIRSQEEQDFLTKHSNKRGSWIGLRDLDIEGEFIWMDSSPLGYSNWQLGEPNDAGQGENCVMMQGS-GQWNDVFCGSYLNSWVCDRLATC
Camelus dromedarius (Arabian camel)	YYFGEGA-KKWIQARFACSNLHGRLVSIHSQEEQDFLTKHANKRGSWIGLRDLDIEGEFIWMDKKPLNYSNWQPGEPNDAGQGENCVMMQVS-GQWNDAFCNNRLDGWVCDRLATC
Camelus bactrianus (Bactrian camel)	YYFGEGA-KKWIQARFACSNLHGRLVSIHSQEEQDFLTKHANKRGSWIGLRDLDIEGEFIWMDKKPLNYSNWQPGEPNDAGQGENCVMMQAS-GQWNDAFCNNRLDGWVCDRLATC
Camelus ferus (wild Bactrian camel)	YYFGEGA-KKWIQARFACSNLHGRLVSIHSQEEQDFLTKHANKRGSWIGLRDLDIEGEFIWMDKKPLNYSNWQPGEPNDAGQGENCVMMQAS-GQWNDAFCNNRLDGWVCDRLATC
Vicugna pacos (alpaca)	YYFGEGA-KKWIQARFACSDLHGRLVSIHSQEEQDFLTKHANKRGSWIGLRDLDIEGEFIWMDKKPLNYSNWQPGEPNDAGQGENCVMMQAS-GKWNDAFCNNRLDGWVCDRLAAC
Odocoileus virginianus texanus (white-tailed deer	YYFGEGS-KKWLEARYTCENLHGRLVSIHSPEEQDFLTKRANWRGSWIGLRDLDIEGEFIWMDNQPLDYSNWQPGEPNDAGLGENCVMMLSS-GKWNDAFCGSKLHGWVCDRLATC
Sus scrofa (pig)	YYFGETA-KKWIQAKYACSSLQGRLVSIHSQEEQDFLTKHAHIRGSWIGLRDLDIEGEFIWMDEKPLDYSNWQPGEPNDAGQGEHCVMMQAS-GQWNDAFCGSYLD-WVCERLATC
Panthera pardus (leopard)	YYFGEGA-KKWIQARFACSKLQGRLVSIHSQEEQDFLAKRANRKGTWIGLRDLDIEGEFIWMDENPLNYSNWRPGEPNNQGQGEDCVMMQGS-GQWNDAFCGSRLDGWVCDRLATC
Felis catus (domestic cat)	YYFGEGA-KKWIQARFACSKLQGRLVSIHSQEEQDFLAKRANRKGTWIGLRDLDIEGEFIWMDENPLNYSNWRPGEPNNQGQGEDCVMMQGS-GQWNDAFCGSRLDGWVCDRLATC
Acinonyx jubatus (cheetah)	YYFGEGA-KKWIQARFACSKLQGRLVSIHSQEEQDFLAKRANRKGTWIGLRDLDIEGEFIWMDENPLNYSNWRPGEPNNQGQGEDCVMMQGS-GQWNDAFCGSRLDGWVCDRLATC
Neomonachus schauinslandi (Hawaiian monk seal)	YYFGEGP-KKWIQARFACGKLQGRLVSIHSQEEQDFLARHANRKGTWIGLRDLDIEGEFIWMDQGPLNYSNWRPGEPNNGGQGEDCVMMQGS-GEWNDAFCGSRLDGWVCDQLATC
Callorhinus ursinus (northern fur seal)	YYFGEGP-KRWIQARFACSKLQGRLVSIHSQEEQDFLVRHANRKGTWIGLRDLDIEGEFIWMDQGPLNYSNWRPGEPNNGGQGEDCVMMQGS-GEWNDAFCGSRLDGWVCDRLATC
Zalophus californianus (California sea lion)	YYFGEGP-KRWIQARFACSKLRGRLVSIHSQEEQDFLVRHANRKGTWIGLRDLDIEGEFIWMDQGPLNYSNWRPGEPNNGGQGEDCVMMQGS-GEWNDAFCGSRLDGWVCDRLATC
Eumetopias jubatus (Steller sea lion)	YYFGEGP-KRWIQARFACSKLRGRLVSIHSQEEQDFLVRHANRKGTWIGLRDLDIEGEFIWMDQGPLNYSNWRPGEPNNGGQGEDCVMMQGS-GEWNDAFCGSRLDGWVCDRLATC
Ailuropoda melanoleuca (Giant Panda)	YYFGKDP-KKWIQARFACSKLQGRLVSIHSEEEQDFLAKRANRKGTWIGLRDLDIEGEFIWMDKDPLNYSNWRPGEPNNGGQGEDCVMMQGS-GQWNDAFCGSQLDGWVCERLATC
Ursus arctos horribilis (Grizzly bear)	YYFGKDP-KKWIQARFACSKLQGRLVSIHSEEEQDFLAKRANRKGTWIGLRDLDIEGEFIWMDKDPLNYSNWRPGEPNNGGQGEDCVMMQGS-GQWNDAFCGSHLDGWVCERLATC
Canis lupus familiaris (dog)	YYFGEEP-KKWIQARFACSKLQGRLASIHSQEEQDFLARYANKKGTWIGLRDLDIEGEFIWMDENPLNYSNWRPGEPNNGGQGEDCVMMQGS-GQWNDAFCGSSLDGWVCDRLATC
Canis lupus dingo (dingo)	YYFGEEP-KKWIQARFACSKLQGRLASIHSQEEQDFLARYANKKGTWIGLRDLDIEGEFIWMDENPLNYSNWRPGEPNNGGQGEDCVMMQGS-GQWNDAFCGSSLDGWVCDRLATC
Vulpes vulpes (red fox)	YYFGEEP-KRWIQARFACSKLQGRLASIHSQEEQDFLARYANKKGTWIGLRDLDIEGEFIWMDENPLNYSNWRPGEPNNGGQGEDCVMMQGS-GQWNDAFCGSSLDGWVCDRLATC
Odobenus rosmarus divergens (Pacific walrus)	YYFGEGP-KRWIQARFACSKLQGRLVSIHSQEEQDFLARHANRKGTWIGLRDLDIEGEFIWMDQGPLNYSNWRPGEPNNGGQGEDCVMMQGS-GEWNDAFCRSRLDGWVCDRLATC
Myotis davidii (David's myotis)	YYFGEGA-KRWIQARNACSGLRGRLVSIHSQEEQDFLATHSNKKGSWIGLRDLHTEGEFVWMDQSALDYSNWRPGEPNNGGQGEDCVMLLGS-GQWNDASCSSYLDGWVCDRLATC
Myotis brandtii (Brandt's bat)	YYFGEGA-KRWIQARNTCRGLRGRLVSIHSQEEQDFLATHSNKKGSWIGLRDLHTEGEFVWMDESPLDYSNWRPGEPNNGGQGEDCVMLLGS-GQWNDAFCSSYLYGWVCDRLATC
Miniopterus natalensis (Natal long-fingered bat)	YYFGEGA-KRWIQARYACSELQGRLVSIHNQEEQDFLAKYINKKGSWIGLRDLNTEGEFVWMDENPLDYSNWRPGEPNNGDQGEDCVMMLGS-GQWNDAFCNSYLDGWVCDRLATC
Eptesicus fuscus (big brown bat)	YYFGEGA-KRWFQARQACRRLRGRLVSIHSQEEQDFLAKHSNKKGSWIGLRDLDREGEFVWMDQRPLDYSNWRPGEPNNGAQGEDCVMLLGS-GQWNDAFCGSYLDGWVCDQLATC
Pteropus alecto (black flying fox)	YYFGKGA-KRWIQAREACSKLQGRLVSIHSQEEQDFLAKYINRQGSWIGLRDLNIEGEFVWMDQKPLNYSNWRPGEPNNGDQGEDCVMMLGF-GQWNDAFCNNYLDGWVCDRLATC
Rousettus aegyptiacus (Egyptian rousette)	YYFGEGA-KRWIQAREACSKLQGRLVSIHSQEEQDFLAKYINRKGSWIGLRDLDIEGQFVWMDQKPLTYSNWRPGEPNNGDQGEDCVMMLGF-GQWNDAFCNNYLDGWVCDRLATC
Desmodus rotundus (common vampire bat)	YYFGEGA-KRWIQARYACNKLNGRLVSIHNQEEQDFLAKYVNKKGSWIGLRDLDIEGEFVWMDGSPLDYSNWRPGEPNNGGEGEDCIMMLGL-GQWNDAFCNSHLEGWICDRLAMC

Hipposideros armiger (great roundleaf bat)	YYFGEGA-KRWIQARLACSKLQGRLVSIHSQEEQDFLVKHINRKESWIGLRDLNIEGNFVWMDENPVNYSNWRPGEPNNGGEGEDCVMMLSS-GQWNDAFCNSLLNGWVCERLATC
Phyllostomus discolor (pale spear-nosed bat)	YYFGEGA-KRWIQARFACSKLHGRLVSIHNQEEQDFLAKYVNKKGSWIGLRDLDIEGEFVWMDGSPLEYSNWRPGEPNNGGEGEDCVMMLGL-GQWNDAFCNSYLEGWMCDRLATC
Equus caballus (horse)	YYFGEGP-KRWIQARFACSKLHGRLVSIHSQEEQDFLTRHANKKGSWIGLRDLNIEGEFVWMDQNPLDYSNWQPGEPNNGGQGEDCVMMRAS-GHWNDAFCGSYLDGWVCDRLVTC
Equus asinus (ass)	YYFGEGP-KRWIQARFACSKLHGRLVSIHSQEEQDFLTRHANKKGSWIGLRDLNIEGEFVWMDQNPLDYSNWQPGEPNNGGQGEDCVMMRAS-GHWNDAFCGSYLDGWVCDRLVTC
Ceratotherium simum simum (southern white rhinoceros)	YYFGEGP-KKWIEARFACSKLQGRLVSIHSQEEQDFLTKRANKKGSWIGLRDLNIEGEFVWMDENPLDYSNWQPGEPNNEGEGEDCVMMQGS-GRWNDAFCLSRLDGWVCDRLATC
Condylura cristata (star-nosed mole)	YYFGQGS-KKWVQARNACRGLQGQLVSIHSQQEQDFLTRHVSRTGSWIGLRDLDIEGEFVWMDGNPLDYSNWGPGEPNNGGQGEDCVMMQGS-GQWNDAFCYSPLDSWVCDRLATC
Sorex araneus (European shrew)	YYFGQGSTKKWLQARNECRALQGQLVSIHSQEEQDFLTPHGSKFGTWIGLRDLDLEGQFVWMDENPLDYSNWGPGEPNNQEPSEDCVVMGHS-GMWNDVDCTSRLDSWVCERLATC
Erinaceus europaeus (western European hedgehog)	YYFGQGS-KKWIQARQACRQLQGQLVSIHSQAEQDFLTKRASRTGSWIGLRDLDVEGDFIWMDEQPLNYSNWRPGEPNNASPGEHCVMMQGS-GQWNDASCRSLLAGWVCDRLATC
Oryctolagus cuniculus (rabbit)	YYFGEGS-KQWIHARYACDNLQGRLVSIHSQEEQDFLAKHVSKRGSWIGLRDLDIEGEFIWVDGSRMDYSNWHPGEPNNGGQGEDCVMMQGS-GLWNDAFCRSLLDAWVCERLATCGPRRA
Tupaia chinensis (Chinese tree shrew)	YYFGKGA-KKWIQARYACEDLGGRLVSIHSREEQDFLTTRANKKGSWIGLRDVFVEGEFLWMDGSPVGYSNWYPGEPNNHDRGEDCVLMKES-GEWNDAFCRDYLDTWVCDRLASCEPPTTLQDGF
Dasypus novemcinctus (nine-banded armadillo)	YYFGEGA-KTWIQAKYTCSDLQGRLVSIHSAEEQDFLTKHSSWKGSWIGLRDLDIEGEFIWMDGSPVDYSNWRPGEPNNGGLGEDCVIMLGS-GQWNDGFCRSEQSGWVCDRLATC
Orycteropus afer afer (aardvark)	YYFGEGA-QMWIQAKYACNDLHAQLVSIHSPEEQDFLSRHAKKD-SWTGLQDLDVEGEFIWLDGSPVDYSNWRPGEPNNQDPGENCMMMVGSSGQWNDAFCRSQLEGWVCQQLATC
Loxodonta africana (African savanna elephant)	YYFGDSA-QMWLQAKYTCEDLHGRLVSIHSSEEQEFLRRHIHRKDSWIGLQDLDIEGEFIWSDGSPMDYSNWRPGEPNDQDQGENCVMILGSSGQWNDAFCRSQLEGWVCEQLATC
Trichechus manatus latirostris (Florida manatee)	YYFGEGS-RMWLQAKYACEDLHGRLVSIHSSEEQNFLNRRAKKD-SWISLQDLDIEGKFIWSDESPVDYSNWRPGEPNNQDPGENCVMMLGSSGQWNDAFCRSQLDGWVCEQLATC
Elephantulus edwardii (cape elephant shrew)	YYFGEGA-QTWLQAKYACEDLHGRLVSIHSSEEQAFLSSHANKEGSWTGLQDLDEEGVFTWLDGSSVDYSNWRPGEPNNQAQGENCVMMLGPSGHWNDAYCRSKLNSWVCERLATC
Echinops telfairi (small Madagascar hedgehog)	YYFGRSKPRKWIQAKHACSDLQGRLVSIHSSQEQDFLSRHASKKGSWIGLQDLDREGEFVWPDGSPVDYSNWRPGEPNDQGLGENCVMLLGTSGQWNDASCRQPLDGWVCERLATC
Chrysochloris asiatica (cape golden mole)	YYFGKDS-WTWLQAKHACSELRGRLVSIHSSEEQDFLRRCIKEKDSWTGLQDLDMEGEFVWLDGSPMDYSNWQVGEPNNQYQGEDCMMMLGSSGQWNDVSCYSELNSWVCERLATC
Galeopterus variegatus (Sunda flying lemur)	YYFGEGI-KRWLHAQYACSDIEGRLVSIHSPEEQDFLTKHANRRGSWIGLHDVDAEGEFIWMDGSAVDYSNWHPGEPNNHGQGEDCVMMQGS-GQWNDAFCRSELGAWVCERLATCGPPATSASAGPPGGPPAHPLHVPPAP
Monodelphis domestica (gray short-tailed opossum)	YFFGKEP-KTWAQAKYACINLQGRLVSIKSREEQVFLNRNANKKGSWIGLRDLDIEGIFLWMDGSSLNYTNWGRGEPNNQGQGEDCVAMRGTSGLWNDANCRGQQDSWICEKLATC
Phascolarctos cinereus (koala)	YFFGKEP-KTWSQAKFACINLQGRLVSIKSKEEQDFLTQRANKKGSWIGLRDLDIEGEFIWMDGSPLNYINWSPGEPNNQGQGEDCVMMRANNGRWNDAYCRGQQDSWICEKLATC
Vombatus ursinus (common wombat)	YFFGKES-KTWSQAKFACINLQGRLVSIKSKEEQAFLTQKASQKGSWIGLRDLDTEGEFTWVDGSPLTYSNWSPGEPNNEGEGEDCVAMRAYNGRWNDAYCRGQQDSWICEKPATC
Sarcophilus harissii (Tasmanian devil)	YFFGKEP-KTWSQAKFACINLQSRLVSIKSREEQSFLTKNANNKGCWIGLRDLDREGEFQWMDESPLNYTNWSPGEPNNQGQGEDCVAMRPFNGKWNDAYCRGYQDSWICEKLATC

Figure S2. PCR primers for cow CD23 cDNA. PCR primers are indicated in blue. Upper panel shows primers for amplification from spleen cDNA library. Lower panel shows primers for re-amplification from first PCR product. The *red* sequence shows the linker sequence used to insert the fragment into the pT5T expression vector. The *green* sequence shows the biotinylation tag appended to the C-terminus.

```
          lGlyLysLeuTrpIleGluLeuArgAlaAlaAsnGlySerValCysAsn
          gggaaagttgtggatcgagctacgcgctgccaacggctctgtgtgcaac
       5'-gggaaagttgtggatcgagctacg-3'

ThrCysProGluAlaTrpIleTyrPheGlnLysLysCysTyrTyrPheGlyGluGlyAla
acgtgccccgaggcatggatctatttccaaaagaagtgctactacttcggggagggcgcc

LysLysTrpIleGlnAlaArgTyrAlaCysGluAsnLeuHisGlyArgLeuValSerIle
aagaaatggatccaggcccggtacgcctgtgaaaatctgcacgggcggctggttagcatc

HisSerProGluGluGlnAspPheLeuThrLysArgAlaAsnTrpArgGlySerTrpIle
cacagcccagaggagcaggacttcctgaccaaacgtgccaactggaggggctcctggatt

GlyLeuArgAspLeuAspIleGluGlyGluPheIleTrpMetAspAsnGlnProLeuAsp
ggccttcgggacctggacattgaaggggagtttatctggatggacaaccagcccctggac

TyrSerAsnTrpGlnProGlyGluProAsnAspAlaGlyGlnGlyGluAsnCysValMet
tatagcaactggcagccaggggagcccaacgatgcaggccagggtgagaactgcgtgatg

MetLeuGlySerGlyLysTrpAsnAspAlaPheCysGlySerGluLeuHisGlyTrpVal
atgctgggctctgggaagtggaatgacgccttctgtggaagcgaacttcatggctgggtg

CysAspArgLeuAlaThrCys***
tgcgaccggctggccacgtgctgagcgttggcctgccaccttggcc
                 3'-ctcgcaaccggacggtggaaccgg-5'
```

```
                                MetAlaAsnGlySerValCysAsn
          ggatccgatcttggaggatgattaaatggccaacggctctgtgtgcaac
       5'-ggatccgatcttggaggatgattaaatggccaacggctctgtgtgcaac

ThrCysProGluAlaTrpIleTyrPheGlnLysLysCysTyrTyrPheGlyGluGlyAla
acgtgccccgaggcatggatctatttccaaaagaagtgctactacttcggggagggcgcc
acgtgc-3'

LysLysTrpIleGlnAlaArgTyrAlaCysGluAsnLeuHisGlyArgLeuValSerIle
aagaaatggatccaggcccggtacgcctgtgaaaatctgcacgggcggctggttagcatc

HisSerProGluGluGlnAspPheLeuThrLysArgAlaAsnTrpArgGlySerTrpIle
cacagcccagaggagcaggacttcctgaccaaacgtgccaactggaggggctcctggatt

GlyLeuArgAspLeuAspIleGluGlyGluPheIleTrpMetAspAsnGlnProLeuAsp
ggccttcgggacctggacattgaaggggagtttatctggatggacaaccagcccctggac

TyrSerAsnTrpGlnProGlyGluProAsnAspAlaGlyGlnGlyGluAsnCysValMet
tatagcaactggcagccaggggagcccaacgatgcaggccagggtgagaactgcgtgatg

MetLeuGlySerGlyLysTrpAsnAspAlaPheCysGlySerGluLeuHisGlyTrpVal
atgctgggctctgggaagtggaatgacgccttctgtggaagcgaacttcatggctgggtg
                                                      3'-cac

CysAspArgLeuAlaThrCysGlyLeuAsnAspIlePheGluAlaGlnLysIleGluTrp
tgcgaccggctggccacgtgcggcctgaatgacatcttcgaagcacagaaaatcgagtgg
acgctggccgaccggtgcacgccggacttactgtagaagcttcgtgtcttttagctcacc

HisGlu***
catgagtaggaattc
gtactcatccttaag-5'
```

Figure S3. PCR primers for mouse CD23 cDNA. PCR primers are indicated in blue. The red sequence shows the linker sequence used to insert the fragment into the pT5T expression vector. The green sequence shows the biotinylation tag appended to the C-terminus.

```
                                                ***MetAla
                             ggatccgatcttggaggatgattaaatggcc
                          5'-ggatccgatcttggaggatgattaaatggcc

ThrAlaCysAsnIleCysProLysAsnTrpLeuHisPheGlnGlnLysCysTyrTyrPhe
actgcatgcaacatatgtcccaagaactggctccatttccaacagaagtgctactatttt
actgcatgcaacatatgtcccaag

GlyLysGlySerLysGlnTrpIleGlnAlaArgPheAlaCysSerAspLeuGlnGlyArg
ggcaagggctccaagcagtggatccaggccaggttcgcctgcagtgacctgcaagggcga

LeuValSerIleHisSerGlnLysGluGlnAspPheLeuMetGlnHisIleAsnLysLys
ctagtcagcatccacagccaaaaggaacaggacttcctgatgcaacacatcaacaagaag

AspSerTrpIleGlyLeuGlnAspLeuAsnMetGluGlyGluPheValTrpSerAspGly
gattcctggattggcctccaggatctcaatatggagggagagtttgtatggtcggacggg

SerProValGlyTyrSerAsnTrpAsnProGlyGluProAsnAsnGlyGlyGlnGlyGlu
agccctgtgggttatagcaactggaatccaggggagcccaataacgggggccagggtgag

AspCysValMetMetArgGlySerGlyGlnTrpAsnAspAlaPheCysArgSerTyrLeu
gactgtgtgatgatgcggggatccggccagtggaacgacgccttctgccgcagctacttg

AspAlaTrpValCysGluGlnLeuAlaThrCysGluIleLeuAsnAspIlePheGluAla
gatgcatgggtgtgtgagcagctggcaacatgtgagatactgaatgacatcttcgaagca
                                          3'-gacttactgtagaagcttcgt

GlnLysIleGluTrpHisGlu***
cagaaaatcgagtggcatgagtaggaattc
gtcttttagctcaccgtactcatccttaag-5'
```

Figure S4. Sequence of codon-optimized cDNA for the CRD from cow CD23 without biotin tag. Bases highlighted in red were changed from the cow genome sequence to insert and remove restriction sites to allow cloning into the pT5T expression vector.

```
                                    MetAlaAsnGlySerValCysAsn
                                   aatggccaacggctctgtgtgcaac

ThrCysProGluAlaTrpIleTyrPheGlnLysLysCysTyrTyrPheGlyGluGlyAla
acgtgccccgaggcatggatctatttccaaaagaagtgctactacttcggggagggcgcc

LysLysTrpIleGlnAlaArgTyrAlaCysGluAsnLeuHisGlyArgLeuValSerIle
aagaaatggatccaggcccggtacgcctgtgaaaatctgcacgggcggctggttagcatc

HisSerProGluGluGlnAspPheLeuThrLysArgAlaAsnTrpArgGlySerTrpIle
cacagcccagaggagcaggacttcctgaccaaacgtgccaactggaggggctcctggatt

GlyLeuArgAspLeuAspIleGluGlyGluPheIleTrpMetAspAsnGlnProLeuAsp
ggccttcgggacctggacattgaaggggagtttatctggatggacaaccagcccctggac

TyrSerAsnTrpGlnProGlyGluProAsnAspAlaGlyGlnGlyGluAsnCysValMet
tatagcaactggcagccaggggagcccaacgatgcaggccagggtgagaactgcgtgatg

MetLeuGlySerGlyLysTrpAsnAspAlaPheCysGlySerGluLeuHisGlyTrpVal
atgctgggctctgggaagtggaatgacgccttctgtggaagcgaacttcatggctgggtg

CysAspArgLeuAlaThrCys***
tgcgaccggctggcaacgtgctagaattc
```
